# Molecular mechanism of sphingosine-1-phosphate action in Duchenne muscular dystrophy

**DOI:** 10.1242/dmm.013631

**Published:** 2013-09-25

**Authors:** Diem-Hang Nguyen-Tran, Nitai C. Hait, Henrik Sperber, Junlin Qi, Karin Fischer, Nick Ieronimakis, Mario Pantoja, Aislinn Hays, Jeremy Allegood, Morayma Reyes, Sarah Spiegel, Hannele Ruohola-Baker

**Affiliations:** 1 Molecular and Cellular Biology Graduate Program, University of Washington, Seattle, WA 98195-7350, USA.; 2 Institute for Stem Cell and Regenerative Medicine, University of Washington, UW Medicine at South Lake Union, 815 Mercer Street, Seattle, WA 98109-4714, USA.; 3 Department of Biochemistry, University of Washington, Seattle, WA 98195-7350, USA.; 4 Department of Biochemistry and Molecular Biology, Virginia Commonwealth University School of Medicine, Richmond, VA 23298, USA.; 5 Department of Chemistry, University of Washington, Seattle, WA 98195-1700, USA.; 6 Department of Pathology, University of Washington, Seattle, WA 98195-7470, USA.

**Keywords:** HDAC, S1P, THI, *dys*, Dystrophin, *mdx*

## Abstract

Duchenne muscular dystrophy (DMD) is a lethal muscle-wasting disease. Studies in *Drosophila* showed that genetic increase of the levels of the bioactive sphingolipid sphingosine-1-phosphate (S1P) or delivery of 2-acetyl-5-tetrahydroxybutyl imidazole (THI), an S1P lyase inhibitor, suppresses dystrophic muscle degeneration. In the dystrophic mouse (*mdx*), upregulation of S1P by THI increases regeneration and muscle force. S1P can act as a ligand for S1P receptors and as a histone deacetylase (HDAC) inhibitor. Because *Drosophila* has no identified S1P receptors and DMD correlates with increased HDAC2 levels, we tested whether S1P action in muscle involves HDAC inhibition. Here we show that beneficial effects of THI treatment in *mdx* mice correlate with significantly increased nuclear S1P, decreased HDAC activity and increased acetylation of specific histone residues. Importantly, the HDAC2 target microRNA genes *miR-29* and *miR-1* are significantly upregulated, correlating with the downregulation of the miR-29 target *Col1a1* in the diaphragm of THI-treated *mdx* mice. Further gene expression analysis revealed a significant THI-dependent decrease in inflammatory genes and increase in metabolic genes. Accordingly, S1P levels and functional mitochondrial activity are increased after THI treatment of differentiating C2C12 cells. S1P increases the capacity of the muscle cell to use fatty acids as an energy source, suggesting that THI treatment could be beneficial for the maintenance of energy metabolism in *mdx* muscles.

## INTRODUCTION

Duchenne muscular dystrophy (DMD) is a lethal muscle-wasting disease that affects 1 in 3500 males. It results in muscle degeneration and eventual death, with affected individuals suffering from diaphragm defects and heart failure (English and Gibbs, 2006). DMD is caused by mutations in the dystrophin gene coding for dystrophin protein, which is located in the dystrophin-glycoprotein complex (DGC) and has an important role in the integrity and connectivity of muscle cell plasma membrane with the extracellular matrix and the inner cytoskeleton (Lapidos et al., 2004; Barresi and Campbell, 2006; Le Rumeur et al., 2010). Because many signaling molecules are anchored to the sarcolemma via dystrophin (Lai et al., 2004; Li et al., 2010), the lack of dystrophin gene expression in individuals with DMD significantly alters the DGC and results in mislocalization of key signaling components (Brenman et al., 1995).

A genetic suppressor screen in *Drosophila* revealed that mutants that should result in an increase in the bioactive sphingolipid sphingosine-1-phosphate (S1P) suppress dystrophic muscle defects (Kucherenko et al., 2008; Pantoja et al., 2013; Pantoja and Ruohola-Baker, 2013). Furthermore, increasing S1P levels by oral delivery of 2-acetyl-4(5)-tetrahydroxybutyl imidazole (THI), an inhibitor of S1P lyase (which catalyzes the irreversible degradation of S1P), also leads to suppression of dystrophic muscle degeneration in flies (Pantoja et al., 2013). In mice, administration of THI is beneficial in the recovery from acute muscle injury in the *mdx* dystrophic model (Loh et al., 2012; Ieronimakis et al., 2013). Treating *mdx* mice with S1P after acute injury promotes muscle regeneration by increasing satellite cell proliferation and myofiber size. THI also increases muscle fiber size, decreases fibrosis and fat deposition, and significantly increases muscle force (Ieronimakis et al., 2013). This further supports previous findings implicating S1P as a muscle trophic factor involved in muscle repair, satellite cell proliferation and myoblast differentiation (Nagata et al., 2006; Bruni and Donati, 2008; Rapizzi et al., 2008; Bruni and Donati, 2013).

Most of the known S1P functions are mediated by a family of five specific G protein-coupled receptors (GPCRs) termed S1PR1–S1PR5 (Rosen et al., 2009; Maceyka et al., 2012). Indeed, the S1P receptors S1PR1 and S1PR2 have been shown to play a role in the beneficial effect of S1P in *mdx* mice (Loh et al., 2012; Ieronimakis et al., 2013). However, previous studies have shown that S1P also has important actions in the nucleus, where it directly binds to and inhibits the histone deacetylases HDAC1 and HDAC2, regulating histone acetylations and gene expression (Hait et al., 2009). Intriguingly, increased *HDAC2* expression correlates with muscular dystrophies and HDAC inhibitors are beneficial in DMD disease (Minetti et al., 2006; Colussi et al., 2008; Consalvi et al., 2013). Because inhibition or deficiency of S1P lyase was associated with elevated nuclear S1P levels and reduced HDAC activity (Ihlefeld et al., 2012), and *Drosophila* do not express known S1PR orthologs, it was of interest to examine the possibility of a common intracellular action of S1P. Here we show that reducing Rpd3, a *Drosophila* homolog of HDAC2, in dystrophic flies reduced the dystrophic phenotype in wing vein formation. Moreover, we found that increasing nuclear S1P levels in *mdx* mice by using THI to inhibit its degradation decreases HDAC activity and increases histone acetylation, resulting in upregulation of muscle metabolic genes and key microRNAs. Our results also suggest that inhibition of HDACs might be the ancestral function of S1P in muscle.

**TRANSLATIONAL IMPACT**

**Clinical issue**

Duchenne muscular dystrophy (DMD) is a lethal X-linked disease characterized by progressive degeneration of muscle tissue. The disease is caused by mutations in the gene encoding dystrophin, a key component of the dystrophin-glycoprotein complex that maintains muscle cell plasma membrane integrity. A study in *Drosophila* indicated that increased levels of the bioactive lipid sphingosine-1-phosphate (S1P) suppress muscle degeneration in DMD. Moreover, oral delivery of 2-acetyl-5-tetrahydroxybutyl imidazole (THI), an inhibitor of S1P lyase, has a protective effect in dystrophic muscle in *Drosophila*. These findings have been further validated by studies in *mdx* mice (a common murine model of DMD), in which THI administration increases the level of S1P, resulting in an increase in muscle force and fiber size. Collectively, these observations support the view that S1P is a muscle trophic factor involved in muscle cell repair and differentiation. In mammals, S1P can act extracellularly as a ligand for S1P receptors and intracellularly as an inhibitor of the histone deacetylases HDAC1 and HDAC2. Because *Drosophila* does not have orthologs for known S1P receptors, and an increase in HDAC2 has been linked with human DMD, it has been proposed that the beneficial effect of S1P in dystrophic muscle is mediated by HDAC inhibition. However, this hypothesis has not yet been tested.

**Results**

In this study, the authors use *Drosophila* and mouse models of muscular dystrophy to explore the mechanism underlying the protective role of S1P. Firstly, they reduced levels of Rpd3, the *Drosophila* homolog of HDAC1 and HDAC2, in a dystrophin mutant background, and report that this leads to a significant suppression of the disease phenotype. In *mdx* mice, HDAC2 expression and activity were shown to be upregulated in several skeletal muscles. Furthermore, treatment with THI reduced disease pathology in the muscles of *mdx* mice following chronic muscle injury, and this correlated with an increase in nuclear S1P and a reduction in HDAC activity. These effects were reproduced in uninjured *mdx* mice. Interestingly, HDAC inhibition in THI-treated mice was shown to be associated with increased expression of two miRNAs that play a role in skeletal muscle regeneration and reduced dystrophic pathology. By applying microarray-based gene expression analysis in THI-treated mice, the authors demonstrate that delivery of the compound significantly decreases the expression of inflammation-related genes and increases metabolic gene expression. THI treatment also increased mitochondrial function.

**Implications and future directions**

In summary, this work demonstrates that increasing nuclear S1P levels by inhibition of its degradation decreases HDAC activity, resulting in upregulation of microRNAs that positively modulate skeletal muscle regeneration, and genes that enhance energy metabolism in dystrophic muscle. The similar findings in *Drosophila* and mice suggest that inhibition of HDACs might be an evolutionarily conserved function of S1P in muscle tissue. The gene expression profiles after THI treatment support the hypothesis that THI increases metabolism in slow twitch muscles, which might be more resistant to dystrophy. Previous studies have revealed fast-to-slow twitch muscle transition as an important step in delaying disease progression in muscular dystrophy. S1P, in addition to other metabolic regulators, might potentiate this process. A comprehensive understanding of the link between S1P, HDAC activity, mitochondria metabolism and muscle fiber types might be useful for the clinical management of DMD and other muscular dystrophies.

## RESULTS

The *sply* mutation of the *Drosophila* S1P lyase can suppress dystrophic phenotypes (Kucherenko et al., 2008; Pantoja et al., 2013; Pantoja and Ruohola-Baker, 2013). S1P is elevated in mouse embryonic fibroblasts that are null for S1P lyase (*Sgpl1^−/−^*) (Ihlefeld et al., 2012). Similarly, mass spectrometry analysis revealed a dramatic increase in the levels of phosphorylated sphingosine and dihydrosphingosine containing 14 carbons (C_14_S1P and C_14_DHS1P) and with conjugated double bonds at C4,6 (Δ^4,6^-C14-sphingadiene-1-phosphate), as well as their corresponding precursors, in the *Drosophila sply* mutant (supplementary material Fig. S1), whereas levels of phosphorylated C_16_ long-chain bases were much lower and only slightly increased in *sply* mutants (data not shown). These data support the notion that the *sply* mutation suppresses *dys* phenotypes by increasing levels of phosphorylated sphingoid bases (Pantoja et al., 2013). In S1P-lyase-deficient mouse embryonic fibroblasts, elevations of S1P were accompanied by reductions in HDAC activity (Ihlefeld et al., 2012). We therefore wondered whether *sply*-based suppression of *dys* phenotypes might occur through repression of HDAC2. To this end, we reduced Rpd3, a *Drosophila* homolog of HDAC2 (Tea et al., 2010), in dystrophic flies and examined the *dys* posterior cross-vein defect phenotype (Christoforou et al., 2008; Kucherenko et al., 2008; Pantoja et al., 2013). Three types of *Rpd3* mutants were used to reduce HDAC2 levels in flies (*Rpd3^12–37^*, *Rpd3^303^* and *Rpd3^04556^*, which encode a non-active protein, a point mutation with a dominant-negative allele and a hypomorphic allele, respectively) (Tea et al., 2010). Interestingly, reduction of Rpd3 in the *dys* mutant background (*Rpd3^303^/TubGal4:UAS-Dys^C-RNAi^* and *Rpd3^12–37^/TubGal4:UAS-Dys^C-RNAi^*) resulted in significant suppression of the dystrophic posterior cross-vein phenotype (supplementary material Fig. S1B,C), indicating that reduction of HDAC2 activity can partially rescue *dys* mutant wing vein phenotypes.

### HDAC2 expression and activity are upregulated in muscles of *mdx* mice

**Fig. 1:**
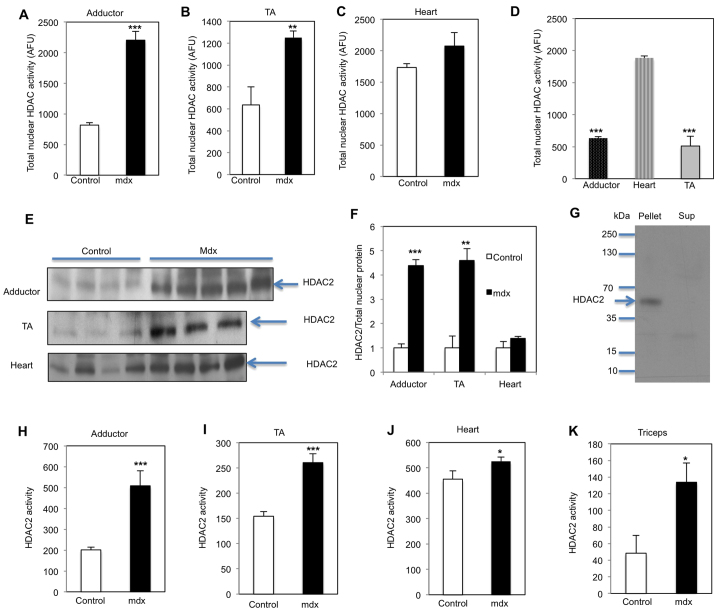
**Total nuclear HDAC activity, and HDAC2 protein level and activity are upregulated in*mdx* mice.** Total nuclear HDAC activity of adductors (A), TA (B) and heart (C) were measured by a fluorescent deacetylation assay in *mdx^4CV^* mice compared with controls (5-month-old males, *n*=5; upregulation in *mdx^4CV^* 2.69- and 1.97-fold in the adductors and TA, respectively). (D) Total nuclear HDAC activity in control mice. (E,F) Western blots of adductor, TA and heart of *mdx^4CV^* mice and controls, analyzed with anti-HDAC2 antibody and quantified normalizing to total nuclear protein (supplementary material Fig. S2; upregulation in *mdx^4CV^* 4.34- and 4.6-fold in the adductors and TA, respectively). (G) Nuclear HDAC2 was immunoprecipitated with anti-HDAC2. The bound proteins were separated by SDS-PAGE and analyzed by western blotting with anti-HDAC2 antibody. (H–K) Immunoprecipitated nuclear HDAC2 activity was determined in adductor (H), TA (I) and heart (J) of *mdx^4CV^* and control mice (upregulation in *mdx^4CV^* 2.52-, 1.95- and 1.15-fold, respectively), and tricep (K) of *mdx* mice compared with control (2.76-fold, 4.5-month-old males, *n*=3); *P<0.05, **P<0.01, ***P<0.001.

Using a fluorescent-based deacetylation assay, in which a fluorophore is generated owing to deacetylation, we show total nuclear HDAC activity was increased by 2.7-fold in *mdx^4CV^* adductor muscles, in agreement with previous studies (Colussi et al., 2008). In addition, HDAC activity was also enhanced in tibialis anterior (TA) muscle (1.97-fold) compared with controls (Fig. 1A,B). Furthermore, HDAC2 protein levels were increased over fourfold in *mdx^4CV^* adductor and TA muscles (Fig. 1E,F; supplementary material Fig. S2). To determine its activity, HDAC2 was immunoprecipitated from muscle nuclear extracts (Fig. 1G) and the activity determined *in vitro* using the fluorescent-based deacetylation assay. HDAC2 activity was significantly increased in both *mdx* adductor and TA muscles compared with controls (2.52- and 1.95-fold, respectively; Fig. 1H,I). In heart tissue from these *mdx^4CV^* mice, the total nuclear HDAC activity and HDAC2 protein levels were only slightly upregulated, and HDAC2 activity was increased 1.15-fold (Fig. 1C,F,J). This lack of robust HDAC increase in *mdx^4CV^* heart tissue might be due to the significantly higher total nuclear HDAC and HDAC2 activity detected in heart compared with other muscles in control animals (Fig. 1D,H–J). HDAC2 activity was also increased 2.7-fold in triceps of *mdx* mice compared with control animals (Fig. 1K). Therefore, total nuclear HDAC activity, HDAC2 protein levels and HDAC2 activity are increased in *mdx* muscles, even in different *dys* mutant genetic backgrounds.

### THI administration in the *mdx* muscle injury model increases muscle S1P levels and reduces muscle HDAC activity

S1P lyase, which catalyzes the irreversible degradation of S1P, can be inhibited *in vivo* by a small molecule, THI, resulting in increased tissue levels of S1P (Schwab et al., 2005; Bagdanoff et al., 2009). In dystrophic mice, treatment with THI after muscle injury has beneficial muscle effects, enhancing satellite-cell-based regeneration as well as significantly increasing muscle fiber size and specific force (Loh et al., 2012; Ieronimakis et al., 2013). In addition, the hallmarks of DMD pathology, fibrosis and fat deposition, are also reduced in THI-treated *mdx* mice. Such regenerative effects were linked to myogenic cell response because intramuscular injection of S1P increases the number of *Myf5^nlacz/+^*-positive myogenic cells and newly regenerated myofibers in injured *mdx* muscles (Ieronimakis et al., 2013).

**Fig. 2:**
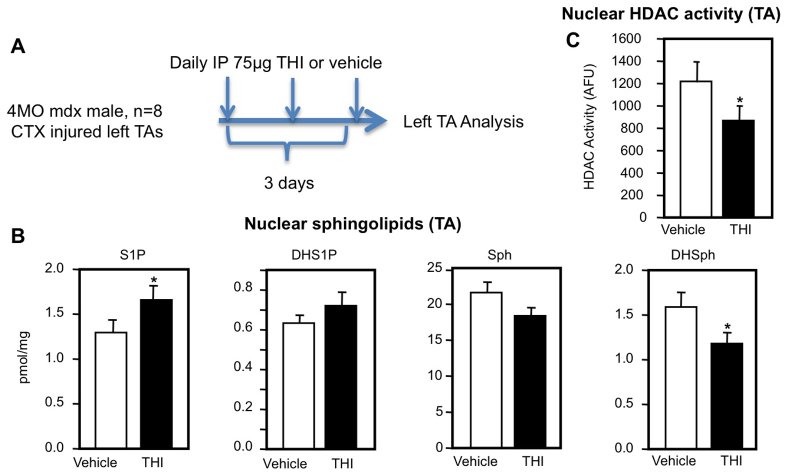
**Increased nuclear S1P and HDAC inhibition in THI-treated*mdx*mice after acute injury.** (A) Experimental scheme. 4-month-old (MO) male *mdx4CV* mice (*n*=8) were injected intraperitoneally (IP) with 75 mg THI or PBS (vehicle) daily for 3 days after CTX injury to TA. (B) TAs were removed, lipids extracted from nuclear fractions and levels of sphingosine-1-phosphate (S1P), dihydrosphingosine-1-phosphate (DHS1P), sphingosine (Sph) and dihydrosphingosine (DHSph) determined by LC-ESI-MS/MS. (C) HDAC activity in nuclear extracts was determined. *P<0.05.

Because S1P has been shown to bind and inhibit HDAC1 and HDAC2, we tested whether THI in these *mdx* injury-model paradigms could increase nuclear S1P and subsequently reduce HDAC activity in muscles. Levels of nuclear S1P were significantly increased in cardiotoxin (CTX)-injured TA muscles following 3 days of treatment of *mdx^4cv^* mice with THI compared with vehicle-treated animals (Fig. 2B). Levels of dihydrosphingosine 1-phosphate (DHS1P), which are much lower than those of S1P, were slightly but not significantly increased. Consistent with the increased nuclear levels of S1P, nuclear HDAC activity was significantly reduced after THI treatment of injured *mdx* mice compared with vehicle controls (Fig. 2C). Taken together with our previous study (Ieronimakis et al., 2013), these data suggest that increased nuclear S1P and reduced HDAC activity correlate with suppressed disease pathology in THI-treated *mdx* mice after acute injury.

### Treatment of non-injured *mdx* mice with THI reduces diaphragm pathology and increases sarcomere integrity

**Fig. 3:**
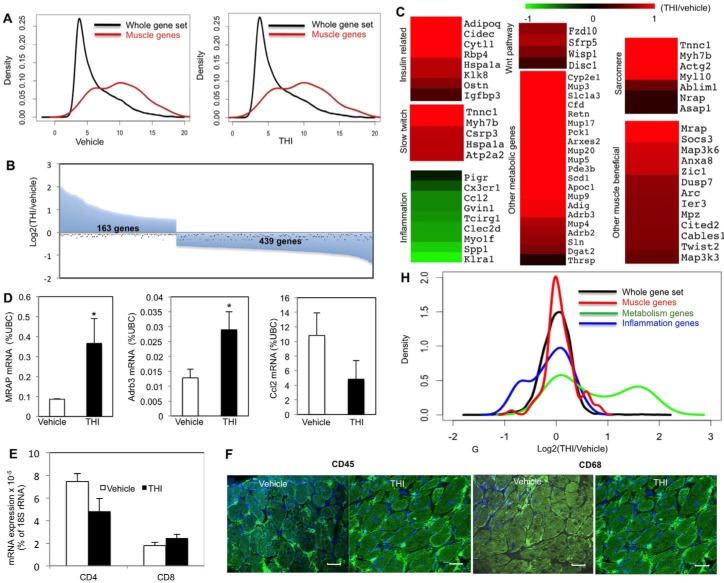
**One-month THI treatment has a beneficial effect in dystrophic pathology in uninjured *mdx* mice.** (A) Experimental scheme. One-month-old (MO) male *mdx* mice were treated daily with THI (50 mg/l THI in 55.5 mM glucose in drinking water, *n*=5) or vehicle (55.5 mM glucose in drinking water, *n*=5) for 1 month. Blood, diaphragm and gastrocnemius were tested for white blood cell count, fibrosis and titin pattern, respectively, to assess THI primary efficacy. Adductors on left sides were used for nuclear protein extraction, sphingolipid measurements, HDAC activity assay and histone acetylation analysis. Right-side adductors were used for RNA extraction and for subsequent microarray and qPCR analyses. (B) White blood cells and lymphocyte counts in THI-treated *mdx* mice before and 24 hours after the withdrawal of THI. (C) H&E and picrosirius red staining of diaphragm collagen from THI- and vehicle-treated *mdx* mice. (D) Quantification of collagen protein in C. (E) Expression of collagen *Col1a1* mRNA was determined by qPCR in diaphragms from vehicle- and THI-treated *mdx* mice (normalized to 18S-rRNA). (F-I) Representative pictures of the Titin sarcomere pattern in gastrocnemius of control and *mdx^4CV^* (3.5 MO) and *mdx* (2 MO) mice treated with vehicle or THI. Titin protein was visualized with the Alexa 488 Fluor (green). Lower picture: high-magnification of one myofibril. (J) Quantification of wild-type Titin sarcomere pattern in control, *mdx^4CV^* (3.5 MO), *mdx* (4 MO) and *mdx* (2 MO) mice treated with vehicle or THI for 1 month. Scale bars: 10 μm (F–I); 50 μm (C). *P<0.05, **P<0.01.

Although a useful experimental strategy, CTX injury is not a physiological representation of chronic muscle injury observed in DMD or *mdx* dystrophic muscles. Therefore, it was of interest to examine the effects of THI in uninjured *mdx* mice. Administration of THI in drinking water for 1 month reduced the white blood cell (WBC) count and lymphocyte levels (Fig. 3B), as expected (Schwab et al., 2005; Ieronimakis et al., 2013). Furthermore, WBC and lymphocyte levels returned to normal 24 hours after THI withdrawal (Fig. 3B), demonstrating the reversibility of its effects. *mdx* mice exhibit strong diaphragm fibrosis, a hallmark of dystrophic muscle pathology, within 1 month of age (Niebroj Dobosz et al., 1997; Hinkle et al., 2007; Ishizaki et al., 2008). We therefore analyzed the fibrosis in the THI-treated and non-treated diaphragm muscles of *mdx* mice after 1 month of treatment. THI treatment decreased the number of collagen fibers, the causal agent of fibrosis, in the diaphragm, as shown by picrosirius red staining (Fig. 3C,D). qPCR revealed that pro-collagen 1a1 (*Col1a1*) mRNA levels were also significantly decreased in THI-treated mice (Fig. 3E). These data suggest that THI has beneficial effects on the dystrophic diaphragm in young *mdx* mice.

We also investigated THI effects on sarcomere molecular architecture. Particularly, to assess myofibril integrity, we examined Titin, a giant elastic protein spanning from the Z-disk to M-line regions of the sarcomere that develops passive force when muscles are stretched (Granzier and Labeit, 2006). Previous studies have shown that DMD pathologies correlate with significant degradation of Titin and abnormal sarcomere pattern of its homolog, Projectin, in the *Drosophila* dystrophic model (Matsumura et al., 1989; Pantoja et al., 2013). This further correlates with the abnormal sarcomere architecture observed previously in the skeletal muscles of *mdx* mice (Friedrich et al., 2010). To test whether THI treatment could have a beneficial effect on Titin sarcomere pattern in *mdx* mice, Titin was visualized by immunostaining using an antibody that recognizes the Titin epitope close to the Z-band, thereby producing a tight banding pattern in each side of the normal Z-disk (Fig. 3F). The integrity of sarcomeres was quantified by counting the number of normal Titin bands, represented as the percentage of wild-type myofibrils. Because DMD is a progressive disease (Blake et al., 2002), the sarcomere architecture in *mdx* mice of different ages was analyzed. This analysis revealed 76.7% of wild-type myofibrils in 2-month-old *mdx*, 57.8% in 3.5-month-old *mdx^4CV^* and 44.3% in 4-month-old *mdx* muscles, compared with 92% in 3.5-month-old normal wild-type muscles (Fig. 3F–H,J; supplementary material Fig. S3A,B). Importantly, in muscles of 2-month-old uninjured *mdx* mice, THI treatment increased the percentage of myofibrils showing a wild-type Titin pattern to 97% (Fig. 3H–J), suggesting that THI has a beneficial effect on sarcomere architecture.

### THI administration in non-injured *mdx* mice increases nuclear S1P levels

**Fig. 4:**
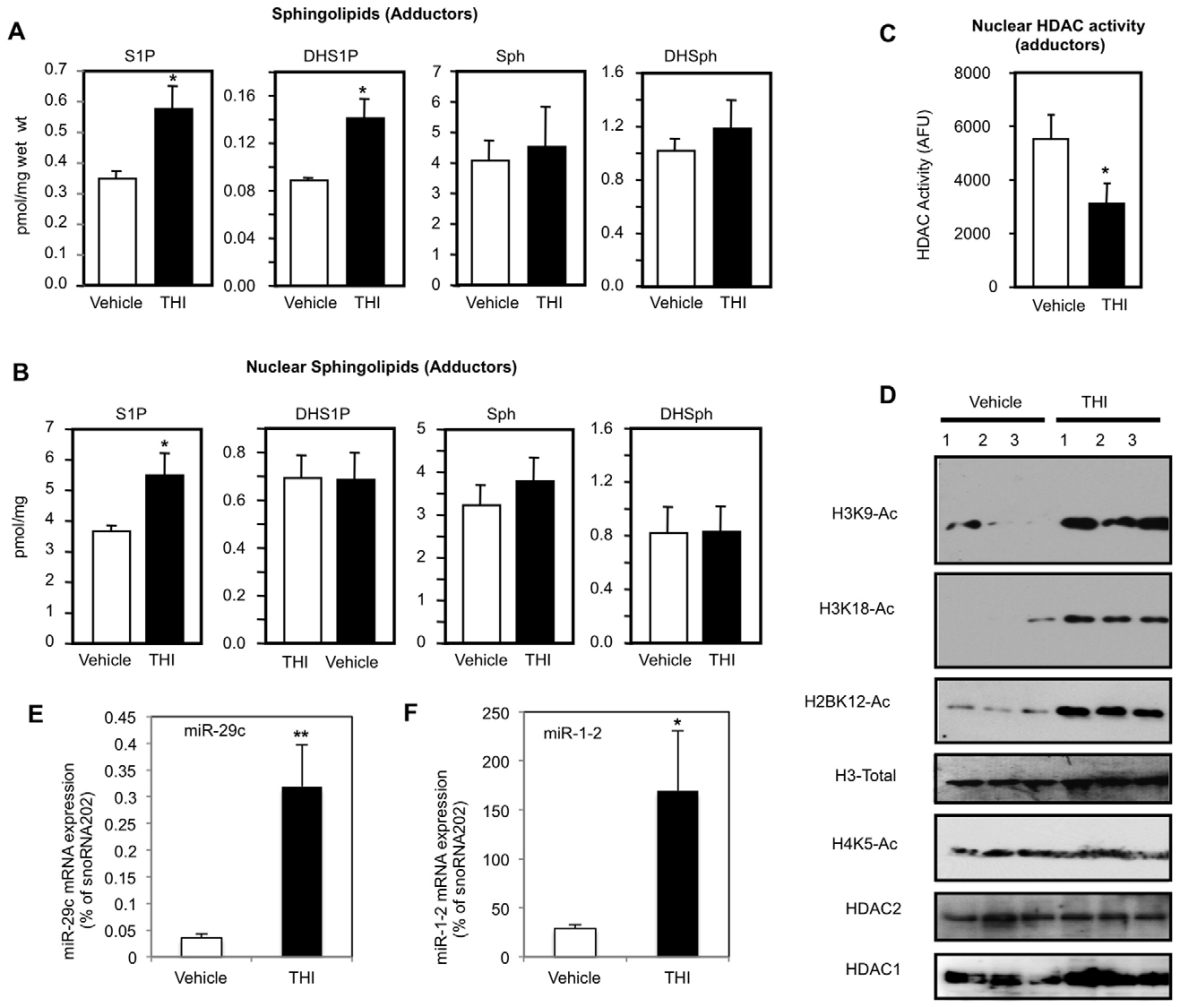
**One-month THI treatment in non-injured *mdx* mice increases muscle and nuclear S1P, decreases nuclear HDAC activity, increases specific histone acetylation and increases HDAC2 target gene expression.** One-month-old male *mdx* mice were treated daily with THI (50 mg/l THI in 55.5 mM glucose in drinking water, *n*=5) or vehicle (55.5 mM glucose in drinking water, *n*=5) for 1 month. (A) Sphingolipid levels in adductor muscles were determined by LC-ESI-MS/MS. (B) Nuclei were isolated and sphingolipid levels determined by mass spectrometry. (C) Nuclear HDAC activity was determined in duplicate samples. (D) Histone acetylations were determined by western blotting with the indicated antibodies. (E,F) qPCR analysis using specific Taqman assays with relative quantification of (E) miR-29c (upregulated ninefold in THI-treated compared with vehicle-treated diaphragms) and (F) miR-1-2 (upregulated 5.9-fold in THI-treated compared with vehicle-treated diaphragms) normalized to endogenous controls of snoRNA202. *P<0.05, **P<0.01.

We analyzed whether the *mdx* mice treated with THI show increased S1P levels in adductor muscles. We observed that S1P and DHS1P levels in these muscles were robustly increased without significant changes in sphingosine or dihydrosphingosine (Fig. 4A). Most importantly, S1P levels in adductor muscle cell nuclei were also significantly increased by THI (Fig. 4B). Thus, the S1P lyase inhibitor THI not only increased muscle S1P levels by reducing degradation of this bioactive lipid but, similar to S1P-lyase-null fibroblasts (Bagdanoff et al., 2009; Ihlefeld et al., 2012), also increased nuclear S1P.

### THI treatment of non-injured *mdx* mice inhibits HDAC activity, and increases histone acetylation and the expression of the HDAC2 targets *miR-29* and *miR-1*

Because S1P is specifically enriched in the nucleus of THI-treated adductor muscles in *mdx* mice, we tested whether it had an inhibitory effect on nuclear HDACs. *In vitro* HDAC assays of nuclear extracts of *mdx* adductor muscles revealed that THI administration reduced HDAC activity by more than 40% (Fig. 4C).

Next, we examined whether decreased HDAC activity correlated with increased histone acetylation *in vivo*. Indeed, THI treatment significantly increased H3K9, H3K18 and H2BK12 acetylation (Fig. 4D), the same histone lysine residues whose acetylations were previously shown to be regulated by nuclear S1P (Hait et al., 2009). Nevertheless, histone H3, HDAC1 and HDAC2 levels were unchanged (Fig. 4D).

In *mdx* mice, HDAC2 is bound to the promoters of *miR-1* and *miR-29*, keeping their expression repressed (Cacchiarelli et al., 2010). Both *miR-29* and *miR-1* have been shown to positively regulate skeletal muscle regeneration, particularly myogenic differentiation (Chen et al., 2006; Wang et al., 2012). In dystrophic *mdx* mouse muscles, miR-29 is significantly downregulated (Wang et al., 2012), possibly affecting the prevalence of fibrosis because miR-29 can reduce fibrosis by targeting the mRNA transcripts of fibrotic genes. To validate the functional relevance of HDAC inhibition by THI treatment, we tested its effects on the expression of these microRNAs. There was a significant increase in expression of both *miR-29* and *miR-1* (9- and 5.6-fold, respectively) in diaphragms from THI-treated *mdx* mice compared with controls (Fig. 4E,F). Taken together, these data suggest that increasing nuclear S1P by treatment with THI inhibits HDAC activity in *mdx* muscles, resulting in upregulation of *miR-1* and *miR-29* and reduction of fibrosis.

### Beneficial muscle genes are upregulated due to a THI-dependent S1P increase

**Fig. 5:**
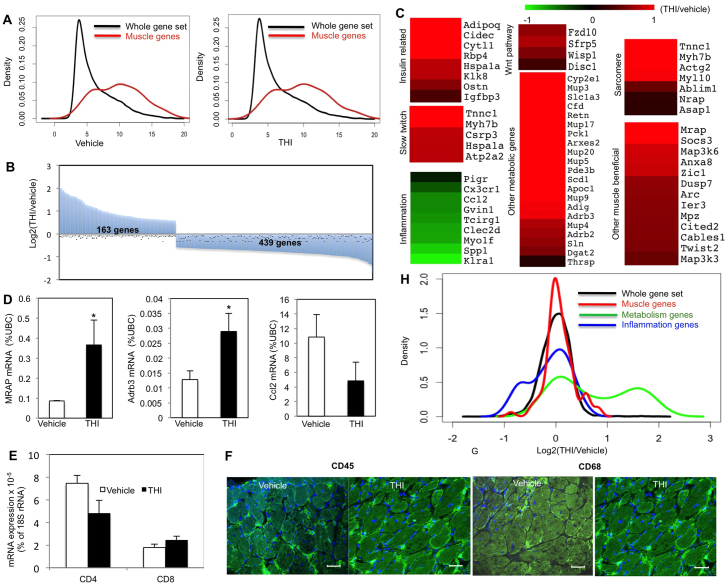
**Beneficial muscle genes, fatty acid metabolism, insulin sensitivity and slow twitch muscle genes are upregulated whereas inflammation genes are downregulated in *mdx* mice treated with THI for 1 month.** One-month-old male *mdx* mice were treated daily with THI (50 mg/l THI in 55.5 mM glucose in drinking water, *n*=5) or vehicle (55.5 mM glucose in drinking water, *n*=5) for 1 month. RNA extracted from the adductors was profiled using microarray analysis. (A) Density plots for gene expression in vehicle- and THI-treated adductor muscles showing enrichment of muscle genes (*P*<2.2e-16 for vehicle- and *P*<2.2e-16 for THI-treated samples). (B) Overview of significant gene expression changes in THI- versus vehicle-treated adductor muscles (163 genes upregulated, 439 downregulated in THI-treated samples). (C) Heatmap presentation of the gene ontology analysis of THI-responsive genes created with the Multi-experiment viewer program. Gene expression data are presented as fold change between THI-treated and control muscles (THI treated/control), and illustrated with green and red color, denoting down- and upregulated genes, respectively. (D) qPCR validation for *Mrap*, *Adrb3* and *Ccl2* gene expression, normalized to the gene expression level of the housekeeping gene, ubiquitin C. *P<0.05. (E) qPCR analysis of inflammation (T cell) markers (*CD4* and *CD8*). (F,G) Immunostaining for CD45 (F) and CD68 (G) in diaphragms after vehicle or THI treatment. (H) Density plot for THI-responsive genes (THI/vehicle gene expression) showing enrichment for muscle and metabolic genes (*P*<0.01909 and *P*<3.211e-10, respectively) and diminished expression of inflammation-related genes (*P*<0.04793). Scale bars: 30 μm.

We next examined global changes in gene expression using microarray analysis. Significant enrichment of muscle genes was revealed by density blot analysis, validating the purity of the adductor muscle samples (Fig. 5A). Furthermore, we identified 602 genes whose expression was significantly changed after THI treatment compared with vehicle treatment (Fig. 5B). Gene ontology analysis by GeneMANIA revealed a decrease in gene expression of chromosome condensation genes as an effect of THI (Table 1), supporting the findings that THI treatment reduced HDAC activity and increased acetylation, affecting the chromosome structure. In addition, expression of fatty-acid-metabolism-related genes was significantly increased in adductors from THI-treated *mdx* mice compared with untreated controls (Table 1).

**Table 1:**
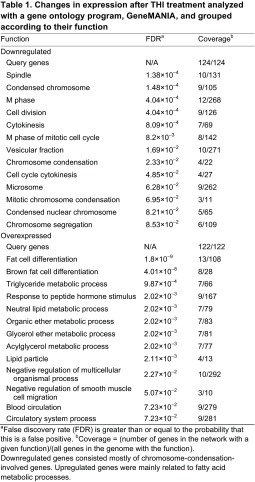
Changes in expression after THI treatment analyzed with a gene ontology program, GeneMANIA, and grouped according to their function

Increased expression of fatty acid metabolism genes might be caused by increased adipocyte infiltration into the muscles tissue or, alternatively, it might indicate a change in myofiber metabolism. To test whether THI treatment increased the number of adipocytes in *mdx* muscles, muscle sections were stained with Oil red O to detect the fat deposits in muscle interstitium. Quantification of the fat deposits within entire cross-sections of diaphragms did not reveal an increase in fat deposits in THI-treated samples (supplementary material Fig. S4A–C), suggesting that the increased fat metabolism gene expression in THI-treated samples is not due to increased adipocyte infiltration to the diaphragm.

Additional functional gene groups that were upregulated in *mdx* muscle after THI treatment were those related to the insulin pathway, slow twitch muscle, metabolism, sarcomeres and the WNT pathway, whereas inflammation-related genes were downregulated, compared with levels in untreated control *mdx* adductor muscles (Fig. 5C). qPCR analysis validated increased expression of *Mrap* and *Adrb3*, representative muscle-beneficial genes that showed a significant increase. The inflammation gene *Ccl2* and the T-cell CD4 marker, but not *CD8*, were somewhat decreased, although not significantly, after THI treatment (Fig. 5D,E). Moreover, no obvious differences compared with controls were observed in the hematopoetic and macrophage populations determined by CD45 and CD68 markers, respectively, in the diaphragms from *mdx* animals treated with THI (Fig. 5F,G; supplementary material Fig. S5).

THI-dependent upregulation of sarcomere-related gene expression (*Myh7b*, *Actg2*, *Myl10*, *AbLim*, *Nrap*, *Asap1* and *Tnnc1*) supports the finding that THI is beneficial for sarcomere architecture (Fig. 3F–I; Fig. 5C) (Roof et al., 1997; Dhume et al., 2006). Similarly, upregulation of genes involved in the WNT pathway supports the finding that THI increases muscle regeneration (Caldow et al., 2011; Reddy et al., 2011; Loh et al., 2012; von Maltzahn et al., 2012; Bentzinger et al., 2013; Ieronimakis et al., 2013; Subramaniam et al., 2013).

The fold-change distributions (THI/vehicle) for genes associated with metabolism, muscle tissue and inflammation are displayed in the form of a density distribution plot (Fig. 5H). When compared with the total data set, a significant increase of metabolic and muscle genes as well as a decrease of inflammation genes is observed compared with the vehicle-treated samples (distribution changes *P-*value based on the Kolmogorov-Smirnov test were 3.211×10^−10^, 0.01909 and 0.04793, respectively; Fig. 5H).

### THI treatment induces metabolic changes

As an energy source for metabolism, slow twitch muscles use mainly fatty acid oxidation, whereas fast twitch muscles utilize glycolysis (Schiaffino and Reggiani, 2011). Interestingly, slow twitch muscles have been shown to be less vulnerable in muscular dystrophy than fast twitch muscles (Webster et al., 1988). Because our density plot analysis showed significant upregulation of fatty acid metabolism genes after THI treatment, we were interested in confirming our microarray data at the functional level. We therefore tested whether THI can alter the metabolism of muscle cells by treating cultured C2C12 myoblasts with THI and analyzing their metabolic flux.

**Fig. 6:**
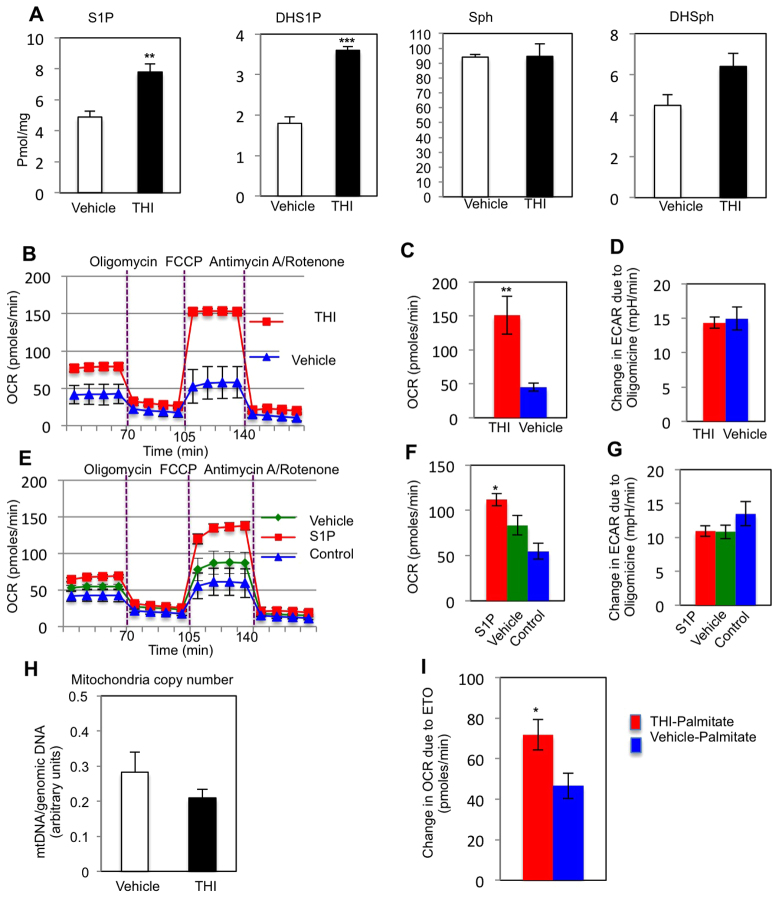
**Intracellular S1P and oxygen consumption is increased in THI-treated differentiating C2C12 cells.** C2C12 cells differentiated for 1 day were treated with 0.05 mg/ml THI or vehicle (1% DMSO) or 0.1 mM S1P-BSA for 1 day, and OCR was recorded with Seahorse apparatus. (A) Cellular S1P and DHS1P were determined by LC-ESI-MS/MS. (B) OCR profile of THI- and vehicle-treated cells. (C,D) Quantification of OCR (C) and ECAR (D) changes after FCCP or oligomycin addition, respectively, in THI- or vehicle-treated C2C12 cells, normalized to the number of cells present in each well, quantified by Hoechst staining (three separate runs, *n*=12 for THI and vehicle). (E) OCR profile in S1P-treated differentiating C2C12 cells. C2C12 cells differentiated for 1 day were treated with 0.1 mM S1P-BSA in serum replacer media (S1P), serum replacer media alone (vehicle) or left untreated in differentiation media (control) for 1 day. (F,G) Quantification of OCR (F) and ECAR (G) change after FCCP or oligomycin addition, respectively, in S1P- or vehicle-treated C2C12 cells, normalized to the number of cells present in each well, quantified by Hoechst staining (two separate runs, *n*=12 for THI and vehicle). (H) Mitochondrial copy number was determined by qPCR in THI- or vehicle-treated differentiating C2C12 cells (*n*=3). The ratio of mtDNA to genomic DNA was calculated by dividing measured copies of *mt-Co1* by copies of *Gapdh*. (I) Fatty-acid-stress assay measuring ETO-responsive OCR change after palmitate addition in THI- or vehicle-treated differentiating C2C12 cells (two separate runs, *n*=9 THI, *n*=12 vehicle). The drugs used: oligomycine (ATP synthase inhibitor), FCCP (ATP uncoupler, stimulates maximum respiration), antimycin A and rotenone (mitochondrial electron transport chain inhibitors), etomoxir (carnitine palmitoyltransferase 1 inhibitor). *P<0.05, **P<0.01, ***P<0.001.

After initiating differentiation for 1 day, C2C12 cells were treated with either THI (0.05 mg/ml) or S1P (0.1 mM) during the second day of differentiation. On the third day the metabolic profiles were examined. As expected, cellular S1P and DHS1P were significantly increased in THI-treated cells (Fig. 6A). To analyze mitochondrial respiration levels, we measured the oxygen consumption rate (OCR), a metabolic parameter representing the mitochondrial respiration levels, using the Seahorse Extracellular Flux analyzer. To record the maximum activity of the electron transport chain uncoupled from ATP synthesis, mitochondrial ATP synthase was inhibited with oligomycine, then the maximum mitochondrial respiration was induced with the proton gradient discharger, carbonyl cyanide 4-(trifluoromethoxy)phenylhydrazone (FCCP). The OCR change was significantly greater after FCCP treatment in THI- and S1P-treated C2C12 cells compared with vehicle-treated samples (Fig. 6B,C,E,F).

Increased mitochondrial respiration could be due to increased mitochondrial copy number or increased mitochondrial activity, for example due to higher efficiency of glucose or fatty acid usage as energy substrates. Utilizing absolute quantification qPCR to analyze the amount of mitochondrial DNA by normalizing to genomic DNA content, we showed no significant difference in the number of mitochondria between THI- and vehicle-treated cells (Fig. 6H), indicating that mitochondrial numbers were not affected by this treatment with THI. To investigate the efficiency of glucose utilization, the extracellular acidification rate (ECAR), which mainly defines the cell glycolysis level, was next determined. We maximized the production of lactic acid by glycolysis by adding the ATP synthase inhibitor oligomycine to inhibit mitochondrial respiration, and analyzed the ECAR changes with and without THI treatment. No significant differences were observed in the maximum ECAR change after adding oligomycine in the mitochondrial stress assay after THI or S1P treatments or in the glucose stress assay for THI- compared with vehicle-treated muscles, suggesting that muscle cells might use substrates other than glucose to obtain the OCR increase observed after THI or S1P treatment (Fig. 6D,G; supplementary material Fig. S6A–D). Interestingly, the fatty acid stress test revealed that the THI-treated cells have greater OCR increases after palmitate addition than the vehicle-treated cells, suggesting that S1P increases the muscle cell capacity to use palmitate as an energy source (Fig. 6I; supplementary material Fig. S7).

## DISCUSSION

**Fig. 7:**
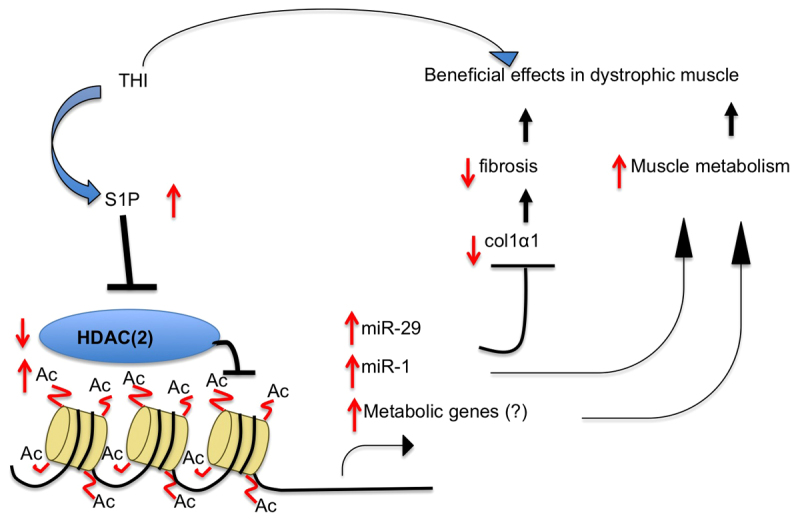
**Model of the intracellular action of S1P to ameliorate DMD phenotypes.** THI treatment leads to increased nuclear S1P levels. Elevated S1P in turn inhibits HDAC2 activity, resulting in increased histone acetylation in the promoter regions of HDAC2 target genes and the upregulation of their expression. The increase in expression of these genes, including *miR-1* and *miR-29*, in turn alleviates fibrosis by decreasing fibrotic gene (*Col1α1*) expression (target of *miR-29*), and increases muscle metabolism (metabolic genes are targets of *miR-1*). Decreased fibrosis and increased muscle metabolism are both beneficial effects on dystrophic muscle.

The molecular mechanisms for the beneficial action of S1P in dystrophic muscle are still not well understood (Ieronimakis et al., 2013; Pantoja et al., 2013; Pantoja and Ruohola-Baker, 2013). We now show that treating CTX-injured *mdx* mice with THI, an S1P lyase inhibitor, increased the level of nuclear S1P and decreased nuclear HDAC activity in the injured *mdx* muscles. THI treatment in uninjured *mdx* mice resulted in decreased diaphragm fibrosis and increased sarcomere structural integrity. These beneficial effects of THI correlate with increased nuclear S1P levels in the affected muscles, decreased nuclear HDAC activity and increased specific histone acetylation marks, resulting in upregulation of HDAC2 target genes *miR-29* and *miR-1*. Further gene expression microarray-based analysis showed a significant decrease in inflammation genes and increase in metabolic genes, especially genes involved in fatty acid metabolism in muscles from THI-treated *mdx* mice. THI treatment of C2C12 cells significantly increased nuclear S1P levels and upregulated mitochondrial respiration. The increased mitochondrial respiration was not a result of increased mitochondrial number but rather of increased fatty acid oxidation efficiency. Hence, muscle beneficial effects of THI treatment both in uninjured and injured *mdx* mice correlate with increased nuclear S1P and reduced HDAC activity levels. Because previous studies suggest a special contribution of HDAC2 in the pathogenesis of DMD (Colussi et al., 2008; Cacchiarelli et al., 2010) and S1P directly binds and inhibits HDAC2 (Hait et al., 2009), it is tempting to suggest that S1P exerts its beneficial effects on dystrophic muscles by inhibiting HDAC2 activity, thereby relieving ‘beneficial genes’ (HDAC2 target genes) from repressed states. The increased expression of these genes in turn lessens dystrophic pathologies and induces a shift in muscle metabolism (Fig. 7). Particularly, elevated levels of miR-29 suppress fibrosis by downregulating the fibrotic gene (*Col1a1*) in *mdx* muscles (Cacchiarelli et al., 2010; Wang et al., 2012). In addition, miR-1 has been shown to target metabolic genes, for example *ABCA1* (ATP-binding cassette transporter ABCA1), *PGM* (phosphoglycerate mutase) and *G6PD* (glucose-6-phosphate dehydrogenase) (Cacchiarelli et al., 2010), which might explain the S1P-related effects on metabolism in THI-treated *mdx* muscles and C2C12 cells.

### Nuclear actions of S1P

Although most of the known biological responses of S1P are mediated by binding to five specific cell surface S1PRs, more recently it has been suggested that S1P also has important intracellular actions. It has been demonstrated that S1P formed in the nucleus by sphingosine kinase 2 (*SphK2*) can bind and inhibit HDAC1 and HDAC2 leading to changes in histone acetylations and gene expression (Hait et al., 2009). Moreover, S1P was increased in the nucleus of S1P-lyase-deficient mouse embryonic fibroblasts, leading to decreased HDAC activity, increased H3K9 acetylation and upregulation of expression of *p21*, a cyclin-dependent kinase inhibitor (Ihlefeld et al., 2012). Because these studies were in cultured cells, their significance to physiological or pathological processes *in vivo* was not known. We are now showing that the beneficial effects of THI in *mdx* mice correlate with increased nuclear S1P, decreased HDAC activity, increased histone acetylation, and upregulation of *miR-29*, *miR-1* and many metabolic genes.

Interestingly, in *Drosophila*, mutants that cause elevation of S1P suppress dystrophic muscle defects (Kucherenko et al., 2008; Pantoja et al., 2013; Pantoja and Ruohola-Baker, 2013). Moreover, we have found that reduced levels of Rpd3, a *Drosophila* homolog of mammalian HDAC2, also reduced the dystrophic wing vein formation phenotype. However, *Drosophila* does not express GPCR orthologs of known vertebrate S1P receptors that respond to S1P. Taken together with the observations that muscle beneficial effects of THI treatment in injured and uninjured *mdx* mice correlate with increased nuclear S1P and reduced HDAC activity, it is reasonable to suggest that inhibition of HDACs might be the ancestral function of S1P in muscle. Although it is possible that other *Drosophila* receptors that respond to extracellular S1P will be identified in the future, currently it seems likely that the common mode of function for S1P in dystrophic flies and mice is intracellular, which could be the precedent in evolution.

### S1P, HDAC functions and the correlation with DMD

Increased HDAC2 in dystrophic muscles contributes to some dystrophic phenotypes, because reduction of HDAC2 partially ameliorates DMD phenotypes (Minetti et al., 2006; Consalvi et al., 2013). We examined HDAC2 activity in THI-treated *mdx* muscles by tracking the expression of its target genes, especially *miR-1* and *miR-29*. Previous studies have shown that HDAC2 binds to the promoter regions of *miR-1* and *miR-29* and downregulates their gene expression in *mdx* muscles (Cacchiarelli et al., 2010). *miR-1* can regulate cellular metabolism through G6PD, and *miR-29* downregulates fibrosis through *Col1a1*; both of these processes are affected by THI treatment in *mdx* mice. We observed a significant increase in gene expression of *miR-1* and *miR-29*, along with a reduction in *Col1a1*. This suggests a possibility that S1P exerts its beneficial effects on dystrophic muscles by suppressing HDAC2 activity (Fig. 7).

### HDAC and the DGC

S1P upregulation induced genetically in the *Drosophila* dystrophic model or by direct muscle delivery of S1P or THI treatment of *mdx* dystrophic mice after acute injury significantly increases muscle regeneration, muscle force and fiber size while reducing DMD pathology of fibrosis and fat deposition (Loh et al., 2012; Ieronimakis et al., 2013; Pantoja et al., 2013). How might S1P increases and inhibition of HDAC relate to defects in the DGC observed in *dys* mutant animals? In this regard, nitric oxide (NO), a product of neuronal nitric oxide synthase (nNOS), also reduces HDAC2 activity by s-nitrosylating it and thereby releasing it from the chromatin. nNOS localizes to the normal DGC, but is mislocalized in individuals with DMD because of the defects in the DGC (Brenman et al., 1995), which results in lower NO levels in the nucleus. Lack of NO in the nucleus of *mdx* mice has been shown to increase HDAC2 activity and consequently silence key muscle genes, whereas increased levels of NO can rescue dystrophic phenotypes in *mdx* mice (Colussi et al., 2008; Consalvi et al., 2013). We now show that the suppressive function of S1P in *mdx* correlates with its ability to inhibit HDAC2. One possibility is that the beneficial effects of intracellular S1P generated by THI treatment of *mdx* mice compensates for the loss of NO caused by mislocalization of nNOS because of the defect in the DGC.

### S1P, HDAC and metabolic mechanisms in C2C12 muscle cells

Previous studies have shown that inhibiting class I HDACs leads to upregulation of mitochondrial biogenesis and oxidative metabolism in C2C12 cells and skeletal muscles (Galmozzi et al., 2013). Because HDAC2 is a class I HDAC, and nuclear S1P is a potent HDAC2 inhibitor (Hait et al., 2009), it is possible that the resulting decreased HDAC2 activity allows the expression of mitochondrial biogenesis and oxidative metabolic genes, leading to increased mitochondrial activity. However, further studies are required to define whether intracellular S1P action in this context is combinatorial, because intracellular S1P might also have a beneficial function directly through mitochondrial activity as has been shown previously (Strub et al., 2011).

### Mitochondrial metabolism and muscle function

Previous studies have shown that slow twitch muscles in *mdx* mice are less prone to injury than are fast twitch muscles (Webster et al., 1988; Selsby et al., 2012; von Maltzahn et al., 2012; Galmozzi et al., 2013). The two types of muscle show a dramatic metabolic difference; slow twitch muscles oxidize fatty acids as their main energy source, whereas fast twitch muscles utilize glucose. Because increased nuclear S1P leads to upregulation of expression of fatty acid metabolism genes and increased mitochondrial activity, it might exert its beneficial function on muscular dystrophy by promoting a switch to a slow twitch muscle metabolism. It has been proposed that slow twitch muscles might be more resistant to injury because of expression of specific genes that are beneficial to muscle (Chemello et al., 2011). For example, increased expression of *utrophin*, a beneficial gene for dystrophic muscle, is observed in slow twitch compared with fast twitch muscles (Tinsley et al., 1998; Gramolini et al., 2001; Fairclough et al., 2013). Furthermore, slow twitch muscles are insulin sensitive and thereby might possess a more efficient energy metabolism than fast twitch muscles (Song et al., 1999; Chen et al., 2000). Interestingly, the gene expression profiles after THI treatment revealed upregulation of insulin-pathway-related genes, supporting the hypothesis of slow twitch muscle metabolism. Furthermore, slow twitch muscles might be more resistant to dystrophy owing to their higher efficiency for energy production, because fatty acids are a much higher energy source than glucose. Previous gene expression analysis studies have also revealed fast-to-slow twitch muscle transition as a single common pathway important for muscular dystrophy (Kotelnikova et al., 2012). Elevation of S1P, in addition to other metabolic regulators, might be useful to reverse this process. Thorough understanding of the link between S1P, HDAC activity, mitochondria metabolism and muscle fiber types might provide an important clue for potential clinical applications for the treatment of DMD.

Based on the results presented in this study, we propose that S1P benefits dystrophic muscles by inhibiting HDAC2 activity. This inhibitory effect results in an increased expression of genes that can suppress dystrophic pathology (fibrosis) and regulates changes in muscle metabolism for better energy usage (Fig. 7).

## MATERIALS AND METHODS

### Animal procedure

Experiments involving animals were performed in accordance with the guidelines of and ethical approval from the University of Washington Institutional Animal Care and Use Committee. The mouse *Dystrophin* mutant strains used are *mdx* (C-to-T transition at position 3185, resulting in a premature stop codon) and *mdx^4CV^* (C-to-T transition in exon 53 at position 7916, resulting in a premature stop codon). The fly strains used in this study are: *Oregon-R, w^1118^, Rpd3^12–37^/TM6,Tb, Rpd3^303^/TM6,Tb, Rpd3^04556^/TM6*, *Sply(29682)*, *cn^1^Sply^05091^/CyO, TubGal4:UAS-Dys^C-RNAi^/TM6B,Tb.*

### Mice used for HDAC activity assay

Wild-type control *C57BL/10ScSn* (control) mice were compared with *mdx* on a *C57BL/10ScSn* background (*C57BL/10ScSn-Dmd*^mdx/^*^J^*, referred to as *mdx*; both groups 4.5-month-old males, *n*=3). Triceps were harvested and snap frozen in liquid nitrogen, and total cell lysates were used for HDAC2 activity assays.

Wild-type control *C58BL/6J* (control) mice were compared with *mdx^4CV^* on a *C58BL/6J* background (*B8Ros.Cg-Dmd^mdx-4CV^/J*, referred to as *mdx^4CV^*; both groups 5-month-old males, *n*=5). Adductors, TA, triceps, heart and brain were harvested and snap frozen, and nuclear extracts were prepared for total HDAC and HDAC2 activity assays.

### Mice used for Titin pattern assay

3.5-month-old *mdx^4CV^* and control mice (*n*=3 males in each group) were used, along with 4-month-old *mdx* and 2-month-old vehicle- or THI-treated *mdx* mice (*n*=5 males in each group). Gastrocnemius (gastro) muscles were harvested and used for Titin staining.

### THI treatment of *mdx* mice in the CTX-injury model

In *mdx^4CV^* mice (4-month-old males, *n*=8), the left TAs were injured with 10 μM CTX (Calbiochem, Darmstadt, Germany) from *Naja nigricollis.* Immediately after injury, the mice were injected intraperitoneally (IP) with THI (250 ml of 0.15 mg/ml) in PBS (*n*=4) or with PBS for vehicle control (*n*=4), twice daily (injections 6 hours apart) for 3 days. On day-4 post-injury, the animals were euthanized and the muscles were dissected for analysis. Dissected TAs were snap frozen and utilized for nuclear S1P quantification and HDAC activity assays.

### THI treatment of uninjured *mdx* mice

THI was administered to 4-week-old male *mdx* mice (Jackson Laboratory) as described previously (Schwab et al., 2005). Briefly, the mice were treated with THI (*n*=5, 50 mg/l in 10 g/l glucose, pH 2.8) or vehicle (*n*=5, 10 g/l glucose, pH 2.8) in drinking water for 1 month. The THI and vehicle solutions were made fresh and replaced twice a week. Blood was collected on the last day of THI treatment and a day after the withdrawal. The mice were euthanized 2 days after withdrawal of THI from the drinking water by the avertin and cervical dislocation method. Adductors, gastrocnemius and diaphragm muscles were harvested and adductors snap frozen. The left adductors were utilized to quantify muscle and nuclear S1P levels, nuclear HDAC activity and histone acetylation profiles. RNA was extracted from right-side adductors and utilized for microarrays and qPCR analysis. Diaphragms were frozen in optimal cutting temperature (OCT) compound with liquid-nitrogen-cooled isopentane and sectioned to 8-μm sections for picrosirius red, CD45 and CD68 staining or embedded in paraffin for hematoxylin and eosin (H&E) staining or snap frozen for RNA extraction. Gastrocnemius muscles were kept in EGTA-Ringer solution and used for Titin pattern analysis.

### Peripheral blood cell analysis

Blood was obtained via retro-orbital blood collection using heparinized capillaries and transferred to blood collection tubes containing 1.6 mg/ml EDTA (SARDTEDT, Numbrecht, Germany) for analysis. The WBC and lymphocyte counts were determined with 30 μl per sample using the Hemavet 950FS system (Drew Scientific, Dallas, TX).

### Mouse histology and immunohistochemistry

Picrosirius red with Fast Green and H&E staining were conducted following established protocols (Kiernan, 2008). Briefly, for picrosirius red staining, OCT diaphragm sections were fixed in ice-cold methanol for 5 minutes at 4°C, washed with water and incubated in picrosirius red for 1 hour. Sections were then washed in acidified water, dehydrated in ethanol and cleared with xylene. Fibrosis was quantified as percentage of area stained red within each 20× field analyzed using ImageJ v1.40. For evaluation of fibrosis, the mean values from three separate sections (~200 μm apart in longitudinal distance) were analyzed for each muscle.

For H&E staining, samples embedded in paraffin were cleared with xylene and dehydrated in ethanol. The diaphragm sections were then stained for hematoxylin for 3 minutes followed by rinsing with deionized water, developing in tap water, and destaining with acid ethanol. Eosin staining was then applied, followed by dehydration in ethanol and clearing in xylene.

For CD45 and CD68 staining, OCT diaphragm sections were hydrated by enclosing muscle areas with a hydrophobic pen and submerging the tissue sections in 1%BSA/PBS. Anti-CD45, -CD68 and -IgG (eBioScience) primary antibodies were then added at 1:100, 1:50 and 1:50 dilution, respectively, and incubated for 1.5 hours. The sections were washed with PBS, and incubated with secondary antibodies, Alexa Fluor 488 donkey anti-rat IgG (H^+^L) (Invitrogen, A21208), at 1:500 dilution for 1 hour. After three washes with PBS, sections were counterstained for 10 seconds with one drop of NucBlue Fixed Cells staining (Life Technologies, R37606). Stained tissues were then mounted with one drop of Fluorescence Mounting media (Dako, S3023) and examined with a confocal microscope. CD45-positive cells were counted in total diaphragm sections (*n*=5 for vehicle and *n*=5 for THI). CD68 marker analysis was done by normalizing the number of positive CD68 cells to the number of total muscle cells in the analyzed tissues (*n*=3 for vehicle and *n*=3 for THI).

### Titin pattern analysis in sarcomeres

The protocol for Titin staining was modified from a previously established method (Knight and Trinick, 1982). Briefly, freshly collected gastro were incubated overnight at 4°C in EGTA-Ringer solution (1 mM EGTA, 100 mM NaCl, 2 mM KCl, 2 mM MgCl_2_, 50 mM Tris pH 7.4) and briefly minced with mini-pestles, followed by three washes in Rigor solution (10 mM Na-phosphate buffer pH 7.0, 100 mM NaCl, 2 mM MgCl_2_). Homogenized muscles were then treated with 1% Triton for 15 minutes at room temperature, fixed in 5% paraformaldehyde for 1 hour, and blocked in PBTB [PBT, 0.4% BSA (w/v), 5% normal goat serum (v/v)] before the staining process. Primary anti-Titin antibody (Hybridoma Bank, 9 D10) was used at 1:10 dilution followed by secondary antibody (Alexa Fluor 488 anti-mouse antibody, Invitrogen, A11029, 1:500 dilution) incubation, both at 4°C overnight. Four washes in PBT were used after each round of antibody incubation, with 10× DAPI added in the third wash after secondary antibody incubation. Stained muscles were stored in Glycerol/Prolong (800 ml 80% glycerol/3% NPG 200 ml Prolong Gold) at 4°C and imaging analyzed by confocal microscopy. Ten Titin bands per myofibril, five myofibrils per mouse and three mice per treatment were analyzed.

### Nuclear protein extraction from frozen tissues

Frozen tissues were ground into fine powder in liquid nitrogen and nuclear proteins were extracted as previously described (Hait et al., 2009). Briefly, minced tissues were homogenized in buffer A [10 mM Hepes pH 7.8, 10 mM KCl, 0.1 mM EDTA, 1 mM Na_3_VO_4_, 1 mM DTT, 0.2 mM PMSF, protease inhibitor (Complete Mini EDTA free, Roche)] and incubated on ice for 15 minutes. 0.75% NP-40 was then added to the suspension and the cells lysed by vortexing at high speed for 10 seconds. Nuclei and cytoplasm were separated by centrifugation at 1000 *g* for 3 minutes at 4°C. High salt buffer (20 mM Hepes pH 7.8, 0.4 M NaCl, 1 mM EDTA, 1 mM Na_3_VO_4_, 1 mM DTT and protease inhibitors) was used to resuspend the nuclei. Nuclear protein was extracted by vortexing for 15 seconds and rocking for 15 minutes. After centrifugation at 14,000 *g* for 5 minutes at 4°C, nuclear proteins were collected from the supernatant fraction.

### Total protein lysate extraction

Total protein was extracted with RIPA buffer [50 mM Tris pH 7.4, 150 mM NaCl, 0.1% SDS, 0.5% sodium deoxycholate, 1% Triton X-100, protease inhibitor (Complete Mini EDTA free, Roche)]. Whole muscles were ground in liquid nitrogen, 500–600 ml RIPA buffer was added to lyse the cells, and the supernatant collected after centrifugation at maximum speed for 10 minutes at 4°C. Protein concentration was measured with the Bradford method.

### Western blot analysis

#### HDAC2 protein level

Nuclear protein fractions (method described above) were separated using 4–20% linear gradient SDS-PAGE (Tris-HCl Ready Gel, Bio-Rad, Hercules, CA) and transferred to polyvinylidene fluoride (PVDF) membranes with a wet transfer system (Bio-Rad). Membranes were blocked for 1 hour with Tris-buffered saline with 0.1% (v/v) Tween20 containing 5% (w/v) non-fat dry milk and incubated overnight in primary anti-HDAC2 (H-54) antibody (Santa Cruz, sc-7899, 1:200 in blocking buffer). After washing (three times in TBST buffer), the blots were incubated for 1 hour with the secondary antibody [goat anti-rabbit IgG (H^+^L) HRP conjugate, Bio-Rad, #170-6515]. The signals were detected using an enhanced chemiluminescence kit (Millipore, Billerica, MA) and CL-XPosure Films (Thermo Scientific), and analyzed using ImageJ, normalized to total nuclear protein stained with coomassie blue.

#### Histone acetylation profile

Equal amounts of nuclear extract proteins were separated by SDS-PAGE, transferred to nitrocellulose and incubated with primary antibodies as indicated in the figure legends, including rabbit polyclonal antibodies to: histone H3, and H4K5ac (Millipore); H3K9ac, H3K18ac (Upstate Biotechnology, USA), H2BK12ac (Cell Signaling); HDAC1, HDAC2 (Santa Cruz Biotechnology). Immunopositive bands were visualized by enhanced chemiluminescence using secondary antibodies conjugated with horseradish peroxidase and Super-Signal West Pico chemiluminescent substrate (Pierce) as described (Cocco et al., 1980).

### Measurement of S1P in mouse tissues

The total lysates and nuclear preparations were isolated as described above. Nuclei were washed extensively with PBS. Internal standards were added (0.5 nmol each, Sphingolipid Mixture II/LM-6005, Avanti Polar Lipids), lipids extracted and sphingolipids quantified by liquid chromatography, electrospray ionization-tandem mass spectrometry (LC-ESIMS/MS, 4000 QTRAP, ABI) as described previously (Hait et al., 2009).

### Immunoprecipitation

Nuclear extracts (500 mg protein) were pre-cleared with normal rabbit IgG (2.5 mg) and protein A/G PLUS-Agarose beads (50 ml) and incubated overnight with 5 mg of HDAC2 Ab (Santa Cruz, sc-7899) and incubated over night. Thereafter, protein A/G PLUS-Agarose beads (50 ml) were added and incubated for 2 hours at 4°C. Beads were collected and washed with HDAC assay buffer (4 times 900 ml; 50 mM Tris pH 8. 137 mM NaCl, 2.7 mM KCl and 1 mM MgCl_2_) and used for HDAC2 activity assays. Aliquots of agarose-bound immune complexes were boiled in SDS-PAGE sample buffer, and the released HDAC2 proteins analyzed by western blotting using anti-HDAC2 as primary antibody.

### HDAC activity assays

HDAC activity was determined with a Fluor-de-Lys^®^ HDAC fluorometric activity assay kit (Enzo Life Sciences). HDAC reactions were initiated by adding *Fluor de Lys^®^* Substrate (100 μM) to 50 μg or 40 μg nuclear protein (NE) for total HDAC activity. For HDAC2 activity assays, HDAC2 was first isolated by immunoprecipitation as described above and the immunoprecipitated HDAC2 was subjected to the activity assay by adding *Fluor de Lys^®^* Substrate (100 mM). Samples were incubated with the substrate at 37°C for 20 minutes. The *Fluor de Lys^®^* Developer was added and the mixture was incubated for another 20 minutes at room temperature. HDAC activity levels were expressed as AFU (arbitrary fluorescence unit) with Perkin Elmer Envision Xcite (2104 Multi-label reader) by measuring fluorescence with excitation at 360 nm and emission at 460 nm.

### Microarrays

Adductor muscles from THI- and vehicle-treated *mdx* mice were used to extract RNA. Frozen tissues were ground into fine powder in liquid nitrogen with a mortar and pestle. Total RNA was isolated using TRIzol (Invitrogen), treated with DNaseI (Thermo Scientific EN0521) and converted to cDNAs. The subsequent synthesized Cy3-labeled cRNA were amplified and purified followed by hybridization in microarray with Agilent platform having 60-mer nucleotides and around 60,000 probes, representing around 30,000 genes. Hybridization and scanning were performed in the microarray facility at the Institute for Systems Biology. Any intensity-dependent biases were removed in the data using the normalize.qspline function in the affy Bioconductor package. Log2-fold-change of gene expression between THI-and vehicle-treated *mdx* samples were used and heatmaps were generated using MultiExperiment Viewer software. Density blots were generated with R studio. Gene sets were retrieved from the Broad Institute. Inflammation gene set include: cytokines and inflammatory response; CCR3 signaling in eosinophils; and inflammation gene set selected from biological database. The fatty acid oxidation gene set contains: fatty acid beta oxidation; and reactome mitochondrial fatty acid oxidation and metabolism gene set acquired from biological database. Muscle gene set includes: muscle development (GO:0007517); Biocarta IGF mTOR pathway; reactome muscle contraction; structural constituent of muscle (GO: 0008307); skeletal muscle development (GO: 0007519).

### Real-time polymerase chain reaction (qPCR)

Total RNA was extracted using TriZol (Invitrogen) and treated with DNaseI (Thermo Scientific, EN0521). RNA abundance was determined using a Nanodrop ND-1000 spectrophotometer (Nanodrop Technologies, Wilmington, DE). After a reverse transcription reaction using the Omniscript RT kit (Qiagen, Valencia, CA), Mrap, Adrb3, Ccl2, Ubi, Colα1, CD4, CD8 and 18S rRNA were analyzed by qPCR with SyberGreen master mix (Applied Biosystems, Carlsbad, CA) on an Applied Biosystems 7300 Real-time PCR cycler. All data were normalized to house keeping gene transcript levels (UBC or 18S rRNA). The gene primers used are as follows (all 5′-3′): Mrap fwd and rev: AGACACTGTCGTCAAAGCCACAG and CGCTCTGTCTCCAGGCTCACCAC, respectively. Ccl2: CAGCCCAGCACCAGCACCAG and CAGCAGGTGAGTGGGGCGTTA. Adrb3: GGCCCTCTCTAGTTCCCAG and TAGCCATCAAACCTGTTGAGC. Col1α1: ACGGCTGCACGAGTCACAC and GGCAGGCGGGAGGTCTT (Kenyon et al., 2003). CD4: TGGTTCGGCATGACACTCTC and GGAAGGAGAACTCCGCTGAC. CD8: AGGAGCCGAAAGCGTGTTTG and TCCTGGCGGTGCCATTTTAC. S1PR1: ATCATGGGCTGGAACTGCATCA and CGAGTCCTGACCAAGGAGTAGAT. Ubiquitin C and 18S rRNA were used as house keeping genes, and the primers used for UBC were: CGTCGAGCCCAGTACCACCAAGAAGG for forward and CCCCCATCACACCGAACAAGCACAAG for reverse (Mamo et al., 2007), and for 18S rRNA were: TTGACGGAAGGGCACCACCAG for forward and GCACCACC ACCCACGGAATCG for reverse (Au et al., 2011).

Analysis of miRNAs was performed using specific TaqMan assays (Applied Biosystem). cDNAs were synthesized using Taqman MicroRNA reverse transcription kit. Primers for hsa-mir-29c (cat. number: 4427975), hsa-mir-1-2 (cat. number: 4427975) and endogenous snoRNA202 (lot number: 0910214-0) were purchased from Applied Biosystems.

### THI-treated C2C12 metabolic flux assay

C2C12 cells were seeded onto Seahorse plates at 5000 cells per XF96 well and differentiated in DMEM (Glutamax, Invitrogen), 10% horse serum (Thermo Scientific* HyClone* Donor Equine Serum), 1% insulin cocktail [insulin, transferrin, selenium solution (ITS-G), Gibco/Invitrogen] for 1 day. On day 2, 0.05 mg/ml THI in 1% DMSO, or 1% DMSO only for control, was added to fresh differentiation medium. Seahorse assays were carried out on day 3. At 1 hour before the assay, culture media were exchanged for base media [unbuffered DMEM (Seahorse XF Assay Media) supplemented with sodium pyruvate (Gibco/Invitrogen, 1 mM) and with 25 mM glucose (for MitoStress assay), 25 mM glucose with 0.5 mM carnitine (for palmitate assay), or 2 mM glutamine (for glucose stress assay)]. Injection of substrates and inhibitors were applied during the measurements to achieve final concentrations of 1 μM for 4-(trifluoromethoxy)phenylhydrazone (FCCP; Seahorse Biosciences), 2.5 μM for oligomycin, 2.5 μM for antimycin and for 2.5 μM rotenone for MitoStress assay; 200 mM palmitate or 33 μM BSA, and 50 μM Etomoxir (ETO) for palmitate assay; and 20 mM glucose and 100 mM 2-deoxy-D-glucose (2-DG) for glucose stress assay. In the mitochondrial stress assay, baseline OCR was first measured, then OCR changes in response to injection of oligomycin, FCCP and finally antimycin and rotenone were determined. The OCR values were further normalized to the number of cells present in each well, quantified by the Hoechst staining (HO33342; Sigma-Aldrich) as measured using fluorescence at 355 nm excitation and 460 nm emission. The baseline OCR was defined as the average values measured before oligomycin injection. Changes in OCR or ECAR in response to the addition of substrates and inhibitors were defined as the maximal change after the chemical injection compared with the last OCR/ECAR value before the injection. The reagents were from Sigma, unless otherwise indicated.

### S1P-treated C2C12 metabolic flux assay

C2C12 cells were treated the same way as in the THI experiment, except for the medium used in the S1P treatment day: Dulbecco’s modified Eagle’s medium/Ham’s F-12 medium (DMEM/F12) containing glutamax with 20% knockout serum replacer (SR), 1 mM sodium pyruvate, 0.1 mM nonessential amino acids, 50 U/ml penicillin, 50 μg/ml streptomycin (all from Invitrogen, Carlsbad, CA), 0.1 mM β-mercaptoethanol (Sigma-Aldrich, St Louis, MO), and 4 ng/ml basic fibroblast growth factor (FGF; Peprotech, Rocky Hill, NJ). S1P in BSA (Cayman Chemical) was used at 0.1 mM final concentration. S1P was dissolved in methanol (0.5 mg/ml) and aliquoted, then solvent was evaporated with a stream of nitrogen to deposit a thin film on the inside of the tube. Prior to use, aliquots were resuspended in PBS with 4 mg/ml BSA (fatty acid free) to a concentration of 500 mM.

### Mitochondria copy number assay

C2C12 DNA was extracted using DNazol (Invitrogen 10503-027) following manufacturer’s protocol. Mitochondria copy number in C2C12 cells was measured as previously described (Facucho-Oliveira et al., 2007). Briefly, the primers used were mt-Co1-F 5′-CAGTCTAATGCTTACTCAGC-3′ and mt-Co1-R 5′-GGGCAGTTACGATAACATTG-3′ for mitochondria DNA and Gapdh-F 5′-GGGAAGCCCATCACCATCTTC-3′ and Gapdh-R 5′-AGAGGGGCCATCCACAGTCT-3′ for genomic DNA. The number of copies of mitochondria DNA and genomic DNA were determined according to a standard curve with tenfold dilutions from 10^2^ to 10^7^ copies, and the ratio of mtDNA to genomic DNA for each sample was calculated. A 20 μl reaction with 2.0 ng of DNA extract, 1× SYBR green mix, and 300 nM of each primer were used. Using the 7300 realtime PCR system (Applied Biosystems), the DNAs were amplified by incubating the reaction mixture at 95°C for 10 minutes, 50 cycles at 95°C for 10 seconds, 53°C for 27 seconds for mt-Co1 or 60°C for 27 seconds for Gapdh, and then 72°C for 28 seconds.

### Statistical analysis

All quantitative data were expressed as mean ± s.e.m. Student’s *t*-test, one tail was used and a *P-*value less than 0.05 was considered statistically significant.

## Supplementary Material

Supplementary Material

## References

[b1-0070041] Au C. G. Butler T. L. Sherwood M. C. Egan J. R. North K. N. Winlaw D. S. (2011). Increased connective tissue growth factor associated with cardiac fibrosis in the mdx mouse model of dystrophic cardiomyopathy. Int. J. Exp. Pathol. 92, 57–65 21121985 10.1111/j.1365-2613.2010.00750.x PMC3052757

[b2-0070041] Bagdanoff J. T. Donoviel M. S. Nouraldeen A. Tarver J. Fu Q. Carlsen M. Jessop T. C. Zhang H. Hazelwood J. Nguyen H. (2009). Inhibition of sphingosine-1-phosphate lyase for the treatment of autoimmune disorders. J. Med. Chem. 52, 3941–3953 19489538 10.1021/jm900278w

[b3-0070041] Barresi R. Campbell K. P. (2006). Dystroglycan: from biosynthesis to pathogenesis of human disease. J. Cell Sci. 119, 199–207 16410545 10.1242/jcs.02814

[b4-0070041] Bentzinger C. F. Wang Y. X. von Maltzahn J. Soleimani V. D. Yin H. Rudnicki M. A. (2013). Fibronectin regulates Wnt7a signaling and satellite cell expansion. Cell Stem Cell 12, 75–87 23290138 10.1016/j.stem.2012.09.015 PMC3539137

[b5-0070041] Blake D. J. Weir A. Newey S. E. Davies K. E. (2002). Function and genetics of dystrophin and dystrophin-related proteins in muscle. Physiol. Rev. 82, 291–329 11917091 10.1152/physrev.00028.2001

[b6-0070041] Brenman J. E. Chao D. S. Xia H. Aldape K. Bredt D. S. (1995). Nitric oxide synthase complexed with dystrophin and absent from skeletal muscle sarcolemma in Duchenne muscular dystrophy. Cell 82, 743–752 7545544 10.1016/0092-8674(95)90471-9

[b7-0070041] Bruni P. Donati C. (2008). Pleiotropic effects of sphingolipids in skeletal muscle. Cell. Mol. Life Sci. 65, 3725–3736 18668202 10.1007/s00018-008-8236-6 PMC11131905

[b8-0070041] Bruni P. Donati C. (2013). Role of sphingosine 1-phosphate in skeletal muscle cell biology. Handbook Exp. Pharmacol. 216, 457–467 10.1007/978-3-7091-1511-4_23 23563671

[b9-0070041] Cacchiarelli D. Martone J. Girardi E. Cesana M. Incitti T. Morlando M. Nicoletti C. Santini T. Sthandier O. Barberi L. (2010). MicroRNAs involved in molecular circuitries relevant for the Duchenne muscular dystrophy pathogenesis are controlled by the dystrophin/nNOS pathway. Cell Metab. 12, 341–351 20727829 10.1016/j.cmet.2010.07.008

[b10-0070041] Caldow M. K. Steinberg G. R. Cameron-Smith D. (2011). Impact of SOCS3 overexpression on human skeletal muscle development in vitro. Cytokine 55, 104–109 21478033 10.1016/j.cyto.2011.03.012

[b11-0070041] Chemello F. Bean C. Cancellara P. Laveder P. Reggiani C. Lanfranchi G. (2011). Microgenomic analysis in skeletal muscle: expression signatures of individual fast and slow myofibers. PLoS ONE 6, e16807 21364935 10.1371/journal.pone.0016807 PMC3043066

[b12-0070041] Chen Y. W. Zhao P. Borup R. Hoffman E. P. (2000). Expression profiling in the muscular dystrophies: identification of novel aspects of molecular pathophysiology. J. Cell Biol. 151, 1321–1336 11121445 10.1083/jcb.151.6.1321 PMC2190600

[b13-0070041] Chen J. F. Mandel E. M. Thomson J. M. Wu Q. Callis T. E. Hammond S. M. Conlon F. L. Wang D. Z. (2006). The role of microRNA-1 and microRNA-133 in skeletal muscle proliferation and differentiation. Nat. Genet. 38, 228–233 16380711 10.1038/ng1725 PMC2538576

[b14-0070041] Christoforou C. P. Greer C. E. Challoner B. R. Charizanos D. Ray R. P. (2008). The detached locus encodes Drosophila Dystrophin, which acts with other components of the Dystrophin Associated Protein Complex to influence intercellular signalling in developing wing veins. Dev. Biol. 313, 519–532 18093579 10.1016/j.ydbio.2007.09.044

[b15-0070041] Cocco L. Maraldi N. M. Manzoli F. A. Gilmour R. S. Lang A. (1980). Phospholipid interactions in rat liver nuclear matrix. Biochem. Biophys. Res. Commun. 96, 890–898 6158946 10.1016/0006-291x(80)91439-4

[b16-0070041] Colussi C. Mozzetta C. Gurtner A. Ill B. Rosati J. Straino S. Ragone G. Pescatori M. Zaccagnini G. Antonini A. (2008). HDAC2 blockade by nitric oxide and histone deacetylase inhibitors reveals a common target in Duchenne muscular dystrophy treatment. Proc. Natl. Acad. Sci. USA 105, 19183–19187 19047631 10.1073/pnas.0805514105 PMC2614736

[b17-0070041] Consalvi S. Mozzetta C. Bettica P. Germani M. Fiorentini F. Del Bene F. Rocchetti M. Leoni F. Monzani V. Mascagni P. (2013). Preclinical studies in the mdx mouse model of duchenne muscular dystrophy with the histone deacetylase inhibitor givinostat. Mol. Med. 19, 79–87 23552722 10.2119/molmed.2013.00011 PMC3667212

[b18-0070041] Dhume A. Lu S. Horowits R. (2006). Targeted disruption of N-RAP gene function by RNA interference: a role for N-RAP in myofibril organization. Cell Motil. Cytoskeleton 63, 493–511 16767749 10.1002/cm.20141

[b19-0070041] English K. M. Gibbs J. L. (2006). Cardiac monitoring and treatment for children and adolescents with neuromuscular disorders. Dev. Med. Child Neurol. 48, 231–235 16483403 10.1017/S0012162206000491

[b20-0070041] Facucho-Oliveira J. M. Alderson J. Spikings E. C. Egginton S. St John J. C. (2007). Mitochondrial DNA replication during differentiation of murine embryonic stem cells. J. Cell Sci. 120, 4025–4034 17971411 10.1242/jcs.016972

[b21-0070041] Fairclough R. J. Wood M. J. Davies K. E. (2013). Therapy for Duchenne muscular dystrophy: renewed optimism from genetic approaches. Nat. Rev. Genet. 14, 373–378 23609411 10.1038/nrg3460

[b22-0070041] Friedrich O. Both M. Weber C. Schürmann S. Teichmann M. D. von Wegner F. Fink R. H. Vogel M. Chamberlain J. S. Garbe C. (2010). Microarchitecture is severely compromised but motor protein function is preserved in dystrophic mdx skeletal muscle. Biophys. J. 98, 606–616 20159157 10.1016/j.bpj.2009.11.005 PMC2820646

[b23-0070041] Galmozzi A. Mitro N. Ferrari A. Gers E. Gilardi F. Godio C. Cermenati G. Gualerzi A. Donetti E. Rotili D. (2013). Inhibition of class I histone deacetylases unveils a mitochondrial signature and enhances oxidative metabolism in skeletal muscle and adipose tissue. Diabetes 62, 732–742 23069623 10.2337/db12-0548 PMC3581211

[b24-0070041] Gramolini A. O. Bélanger G. Thompson J. M. Chakkalakal J. V. Jasmin B. J. (2001). Increased expression of utrophin in a slow vs. a fast muscle involves posttranscriptional events. Am. J. Physiol. 281, C1300–C1309 10.1152/ajpcell.2001.281.4.C1300 11546668

[b25-0070041] Granzier H. L. Labeit S. (2006). The giant muscle protein titin is an adjustable molecular spring. Exerc. Sport Sci. Rev. 34, 50–53 16672800 10.1249/00003677-200604000-00002

[b26-0070041] Hait N. C. Allegood J. Maceyka M. Strub G. M. Harikumar K. B. Singh S. K. Luo C. Marmorstein R. Kordula T. Milstien S. (2009). Regulation of histone acetylation in the nucleus by sphingosine-1-phosphate. Science 325, 1254–1257 19729656 10.1126/science.1176709 PMC2850596

[b27-0070041] Hinkle R. T. Lefever F. R. Dolan E. T. Reichart D. L. Dietrich J. A. Gropp K. E. Thacker R. I. Demuth J. P. Stevens P. J. Qu X. A. (2007). Corticortophin releasing factor 2 receptor agonist treatment significantly slows disease progression in mdx mice. BMC Med. 5, 18 17626629 10.1186/1741-7015-5-18 PMC1936998

[b28-0070041] Ieronimakis N. Pantoja M. Hays A. Dosey T. Qi J. Fischer K. Hoofnagle A. Sadilek M. Chamberlain J. Ruohola-Baker H. (2013). Increased Sphingosine-1-Phosphate ameliorates disease pathology in mdx mice after acute injury. Skelet. Muscle 3, 20 23915702 10.1186/2044-5040-3-20 PMC3750760

[b29-0070041] Ihlefeld K. Claas R. F. Koch A. Pfeilschifter J. M. Meyer Zu Heringdorf D. (2012). Evidence for a link between histone deacetylation and Ca^2^+ homoeostasis in sphingosine-1-phosphate lyase-deficient fibroblasts. Biochem. J. 447, 457–464 22908849 10.1042/BJ20120811

[b30-0070041] Ishizaki M. Suga T. Kimura E. Shiota T. Kawano R. Uchida Y. Uchino K. Yamashita S. Maeda Y. Uchino M. (2008). Mdx respiratory impairment following fibrosis of the diaphragm. Neuromuscular Disorders 18, 342–348 18358722 10.1016/j.nmd.2008.02.002

[b31-0070041] Kenyon N. J. Ward R. W. McGrew G. Last J. A. (2003). TGF-beta1 causes airway fibrosis and increased collagen I and III mRNA in mice. Thorax 58, 772–777 12947136 10.1136/thorax.58.9.772 PMC1746790

[b32-0070041] Kiernan J. (2008). Histological and Histochemical Methods: Theory and Practice, 4th edn. Cold Spring Harbor, NY: Cold Spring Harbor Laboratory Press

[b33-0070041] Knight P. J. Trinick J. A. (1982). Preparation of myofibrils. Methods Enzymol. 85B, 9–12 7121291 10.1016/0076-6879(82)85004-0

[b34-0070041] Kotelnikova E. Shkrob M. A. Pyatnitskiy M. A. Ferlini A. Daraselia N. (2012). Novel approach to meta-analysis of microarray datasets reveals muscle remodeling-related drug targets and biomarkers in Duchenne muscular dystrophy. PLOS Comput. Biol. 8, e1002365 22319435 10.1371/journal.pcbi.1002365 PMC3271016

[b35-0070041] Kucherenko M. M. Pantoja M. Yatsenko A. S. Shcherbata H. R. Fischer K. A. Maksymiv D. V. Chernyk Y. I. Ruohola-Baker H. (2008). Genetic modifier screens reveal new components that interact with the Drosophila dystroglycan-dystrophin complex. PLoS ONE 3, e2418 18545683 10.1371/journal.pone.0002418 PMC2398783

[b36-0070041] Lai K. M. Gonzalez M. Poueymirou W. T. Kline W. O. Na E. Zlotchenko E. Stitt T. N. Economides A. N. Yancopoulos G. D. Glass D. J. (2004). Conditional activation of akt in adult skeletal muscle induces rapid hypertrophy. Mol. Cell. Biol. 24, 9295–9304 15485899 10.1128/MCB.24.21.9295-9304.2004 PMC522257

[b37-0070041] Lapidos K. A. Kakkar R. McNally E. M. (2004). The dystrophin glycoprotein complex: signaling strength and integrity for the sarcolemma. Circ. Res. 94, 1023–1031 15117830 10.1161/01.RES.0000126574.61061.25

[b38-0070041] Le Rumeur E. Winder S. J. Hubert J. F. (2010). Dystrophin: more than just the sum of its parts. Biochim. Biophys. Acta 1804, 1713–1722 20472103 10.1016/j.bbapap.2010.05.001

[b39-0070041] Li D. Bareja A. Judge L. Yue Y. Lai Y. Fairclough R. Davies K. E. Chamberlain J. S. Duan D. (2010). Sarcolemmal nNOS anchoring reveals a qualitative difference between dystrophin and utrophin. J. Cell Sci. 123, 2008–2013 20483958 10.1242/jcs.064808 PMC2880012

[b40-0070041] Loh K. C. Leong W. I. Carlson M. E. Oskouian B. Kumar A. Fyrst H. Zhang M. Proia R. L. Hoffman E. P. Saba J. D. (2012). Sphingosine-1-phosphate enhances satellite cell activation in dystrophic muscles through a S1PR2/STAT3 signaling pathway. PLoS ONE 7, e37218 22606352 10.1371/journal.pone.0037218 PMC3351440

[b41-0070041] Maceyka M. Harikumar K. B. Milstien S. Spiegel S. (2012). Sphingosine-1-phosphate signaling and its role in disease. Trends Cell Biol. 22, 50–60 22001186 10.1016/j.tcb.2011.09.003 PMC3253987

[b42-0070041] Mamo S. Gal A. B. Bodo S. Dinnyes A. (2007). Quantitative evaluation and selection of reference genes in mouse oocytes and embryos cultured in vivo and in vitro. BMC Dev. Biol. 7, 14 17341302 10.1186/1471-213X-7-14 PMC1832186

[b43-0070041] Matsumura K. Shimizu T. Nonaka I. Mannen T. (1989). Immunochemical study of connectin (titin) in neuromuscular diseases using a monoclonal antibody: connectin is degraded extensively in Duchenne muscular dystrophy. J. Neurol. Sci. 93, 147–156 2592979 10.1016/0022-510x(89)90185-8

[b44-0070041] Minetti G. C. Colussi C. Adami R. Serra C. Mozzetta C. Parente V. Fortuni S. Straino S. Sampaolesi M. Di Padova M. (2006). Functional and morphological recovery of dystrophic muscles in mice treated with deacetylase inhibitors. Nat. Med. 12, 1147–1150 16980968 10.1038/nm1479

[b45-0070041] Nagata Y. Partridge T. A. Matsuda R. Zammit P. S. (2006). Entry of muscle satellite cells into the cell cycle requires sphingolipid signaling. J. Cell Biol. 174, 245–253 16847102 10.1083/jcb.200605028 PMC2064184

[b46-0070041] Niebroj Dobosz I. Fidzianska A. Glinka Z. (1997). Comparative studies of hind limb and diaphragm muscles of mdx mice. Basic Appl. Myol. 7, 381–386

[b47-0070041] Pantoja M. Ruohola-Baker H. (2013). Drosophila as a starting point for developing therapeutics for the rare disease Dunchenne Muscular Dystrophy. Rare Dis. 1, e24995 10.4161/rdis.24995 PMC3932943 25002997

[b48-0070041] Pantoja M. Fischer K. A. Ieronimakis N. Reyes M. Ruohola-Baker H. (2013). Genetic elevation of sphingosine 1-phosphate suppresses dystrophic muscle phenotypes in Drosophila. Development 140, 136–146 23154413 10.1242/dev.087791 PMC3513996

[b49-0070041] Rapizzi E. Donati C. Cencetti F. Nincheri P. Bruni P. (2008). Sphingosine 1-phosphate differentially regulates proliferation of C2C12 reserve cells and myoblasts. Mol. Cell. Biochem. 314, 193–199 18454302 10.1007/s11010-008-9780-y

[b50-0070041] Reddy V. S. Valente A. J. Delafontaine P. Chandrasekar B. (2011). Interleukin-18/WNT1-inducible signaling pathway protein-1 signaling mediates human saphenous vein smooth muscle cell proliferation. J. Cell. Physiol. 226, 3303–3315 21321938 10.1002/jcp.22676 PMC3111842

[b51-0070041] Roof D. J. Hayes A. Adamian M. Chishti A. H. Li T. (1997). Molecular characterization of abLIM, a novel actin-binding and double zinc finger protein. J. Cell Biol. 138, 575–588 9245787 10.1083/jcb.138.3.575 PMC2141644

[b52-0070041] Rosen H. Gonzalez-Cabrera P. J. Sanna M. G. Brown S. (2009). Sphingosine 1-phosphate receptor signaling. Annu. Rev. Biochem. 78, 743–768 19231986 10.1146/annurev.biochem.78.072407.103733

[b53-0070041] Schiaffino S. Reggiani C. (2011). Fiber types in mammalian skeletal muscles. Physiol. Rev. 91, 1447–1531 22013216 10.1152/physrev.00031.2010

[b54-0070041] Schwab S. R. Pereira J. P. Matloubian M. Xu Y. Huang Y. Cyster J. G. (2005). Lymphocyte sequestration through S1P lyase inhibition and disruption of S1P gradients. Science 309, 1735–1739 16151014 10.1126/science.1113640

[b55-0070041] Selsby J. T. Morine K. J. Pendrak K. Barton E. R. Sweeney H. L. (2012). Rescue of dystrophic skeletal muscle by PGC-1α involves a fast to slow fiber type shift in the mdx mouse. PLoS ONE 7, e30063 22253880 10.1371/journal.pone.0030063 PMC3256197

[b56-0070041] Song X. M. Ryder J. W. Kawano Y. Chibalin A. V. Krook A. Zierath J. R. (1999). Muscle fiber type specificity in insulin signal transduction. Am. J. Physiol. 277, R1690–R1696 10600915 10.1152/ajpregu.1999.277.6.R1690

[b57-0070041] Strub G. M. Paillard M. Liang J. Gomez L. Allegood J. C. Hait N. C. Maceyka M. Price M. M. Chen Q. Simpson D. C. (2011). Sphingosine-1-phosphate produced by sphingosine kinase 2 in mitochondria interacts with prohibitin 2 to regulate complex IV assembly and respiration. FASEB J. 25, 600–612 20959514 10.1096/fj.10-167502 PMC3023391

[b58-0070041] Subramaniam S. Sreenivas P. Cheedipudi S. Reddy V. R. Shashidhara L. S. Chilukoti R. K. Mylavarapu M. Dhawan J. (2013). Distinct transcriptional networks in quiescent myoblasts: a role for Wnt signaling in reversible vs. irreversible arrest. PLoS ONE 8, e65097 23755177 10.1371/journal.pone.0065097 PMC3670900

[b59-0070041] Tea J. S. Chihara T. Luo L. (2010). Histone deacetylase Rpd3 regulates olfactory projection neuron dendrite targeting via the transcription factor Prospero. Neuroscience 30, 9939–9946 20660276 10.1523/JNEUROSCI.1643-10.2010 PMC2924735

[b60-0070041] Tinsley J. Deconinck N. Fisher R. Kahn D. Phelps S. Gillis J. M. Davies K. (1998). Expression of full-length utrophin prevents muscular dystrophy in mdx mice. Nat. Med. 4, 1441–1444 9846586 10.1038/4033

[b61-0070041] von Maltzahn J. Renaud J. M. Parise G. Rudnicki M. A. (2012). Wnt7a treatment ameliorates muscular dystrophy. Proc. Natl. Acad. Sci. USA 109, 20614–20619 23185011 10.1073/pnas.1215765109 PMC3528612

[b62-0070041] Wang L. Zhou L. Jiang P. Lu L. Chen X. Lan H. Guttridge D. C. Sun H. Wang H. (2012). Loss of miR-29 in myoblasts contributes to dystrophic muscle pathogenesis. Mol. Ther. 20, 1222–1233 22434133 10.1038/mt.2012.35 PMC3369280

[b63-0070041] Webster C. Silberstein L. Hays A. P. Blau H. M. (1988). Fast muscle fibers are preferentially affected in Duchenne muscular dystrophy. Cell 52, 503–513 3342447 10.1016/0092-8674(88)90463-1

