# Supplementary material for: Molecular mechanism of sphingosine-1-phosphate action in Duchenne muscular dystrophy
Source: Dis Model Mech. 2013 Sep 25;7(1):41–54. doi: 10.1242/dmm.013631 (PMC3882047; doi:10.1242/dmm.013631)
Supplement: Supplementary Material [file supp_7_1_41__index.html]

Molecular mechanism of sphingosine-1-phosphate action in Duchenne muscular dystrophy — Molecular mechanism of sphingosine-1-phosphate action in Duchenne muscular dystrophy — Supplementary Material 

# Molecular mechanism of sphingosine-1-phosphate action in Duchenne muscular dystrophy

## DMM013631 Supplementary Material

**Files in this Data Supplement:**

- **Supplementary Material PDF**
