# Supplementary material for: Molecular mechanism of sphingosine-1-phosphate action in Duchenne muscular dystrophy
Source: Dis Model Mech. 2013 Sep 25;7(1):41–54. doi: 10.1242/dmm.013631 (PMC3882047; doi:10.1242/dmm.013631)
Supplement: Supplementary Material [file supp_013631_DMM013631.pdf]

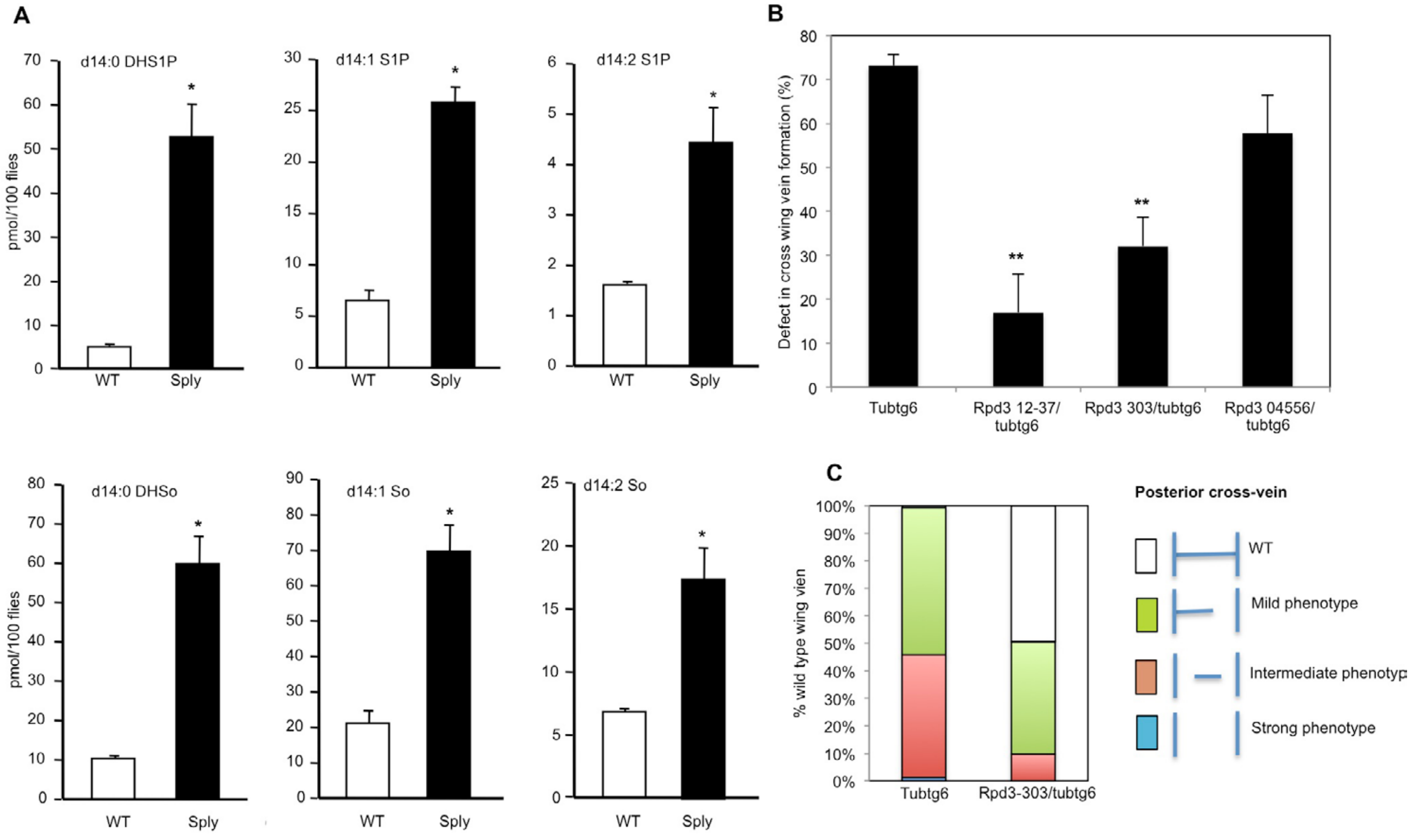


**Fig S1. S1P levels are increased in sply mutant and reduced Rpd3 in dystrophic flies supresses dystrophic phenotype in wing vein formation.** (A) Changes in phosphorylated long chain sphingoid bases in Drosophila *sply* mutant. Lipids were extracted from 100 wild type Drosophila and *Sply* mutants and levels of phosphorylated sphingosine and dihydrosphingosine containing 14 carbons ($C_{14}$S1P and $C_{14}$DHS1P) and conjugated double bonds at C4,6 ($\Delta^{4,6}$-C14-sphingadiene-1-phosphate), and precursors were determined by LC-ESI-MS/MS. (B, C) Reducing HDAC2 in dystrophic flies suppresses the dystrophic wing vein formation phenotype. Dystrophic (*dys* KD) flies, *TubGal4:UAS-Dys*$^{C\text{-}RNAi}$(*tubtg6*) with reduced Rpd3, *Rpd3*$^{12\text{-}37}$*/tubtg6, Rpd3*$^{303}$*/tubtg6, Rpd3*$^{04556}$*/tubtg6*. (B) Percent of defective posterior cross-veins in wings of *dys* KD animals (*tubtg6*) with and without Rpd3 reduction. (C) Posterior cross-vein phenotypes observed in *dys* KD animals (*tubtg6*) with and without reduced Rpd3 (*Rpd3*$^{303}$*/tubtg6*).

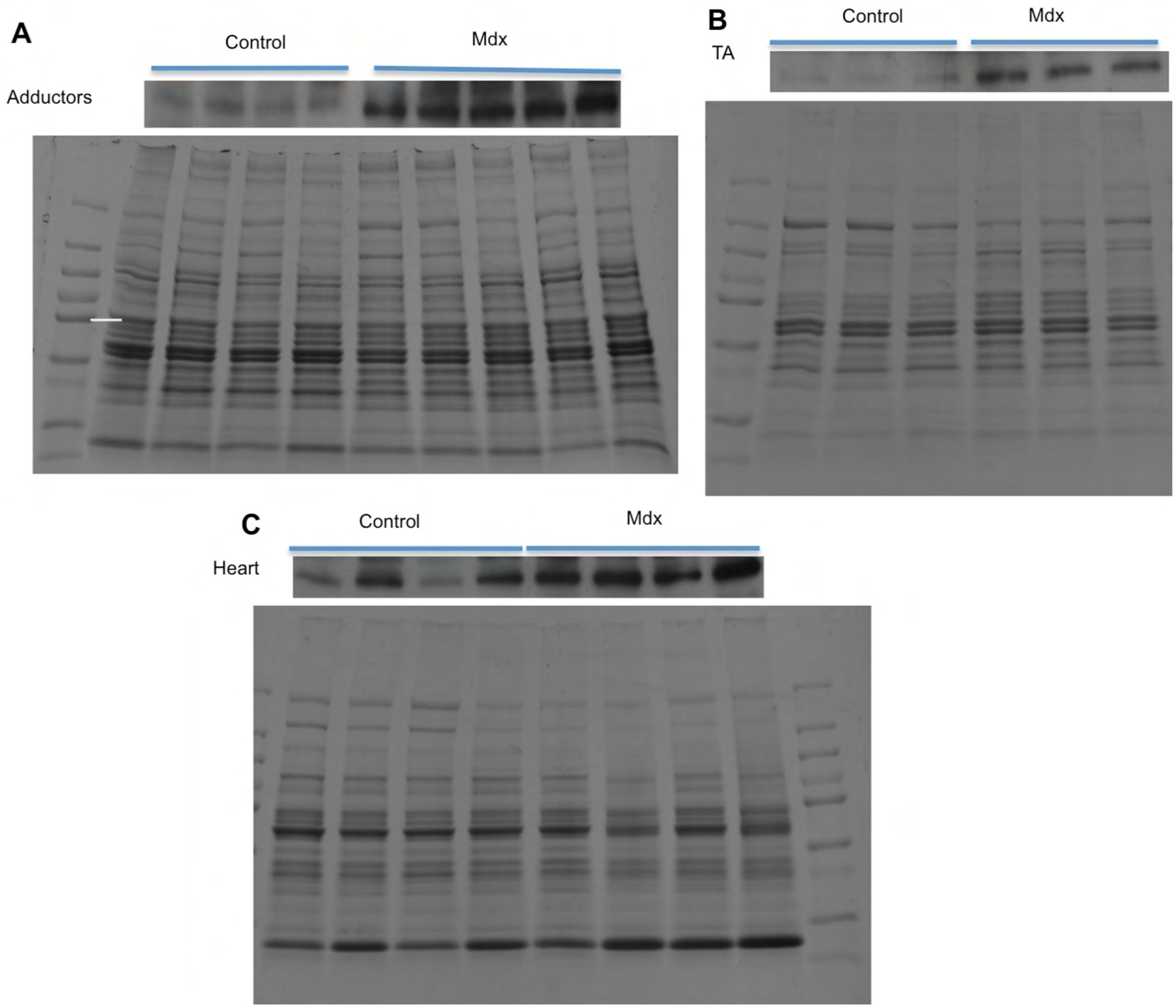


**Fig S2. Coomassie blue staining control for Fig. 1 normalization.** 30µg nuclear protein of *mdx*$^{4CV}$ or control muscles was run for each lane, same amount as used in protein gels of Fig. 1 (anti-HDAC2 western from Fig. 1E is shown again above the lanes). (A) Adductors, (B) TAs, (C) Heart.

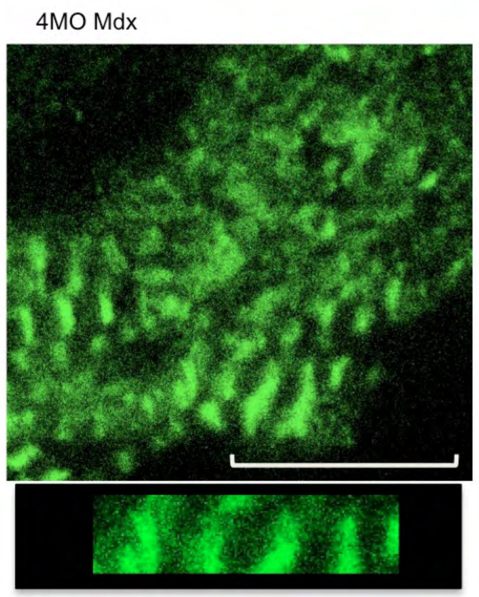


**Fig S3. Titin pattern in 4MO *mdx* muscles.** Scale bar: 10 µm.

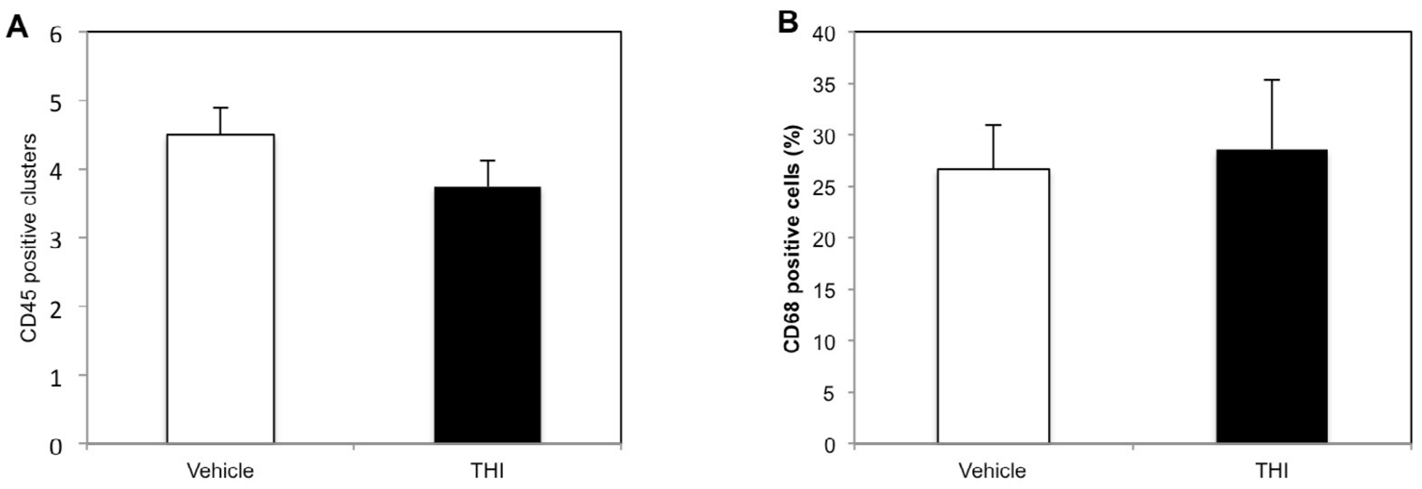


**Fig S4. Fat infiltration in diaphragms is not increased after 1 month THI treatment compared to vehicle treated *mdx* mice**. Representative Oil Red O staining in (A) vehicle and (B) THI treated diaphragms. Quantification of fat area normalized to muscle area show a trend of decrease in fat infiltration in THI compared to vehicle treated samples.

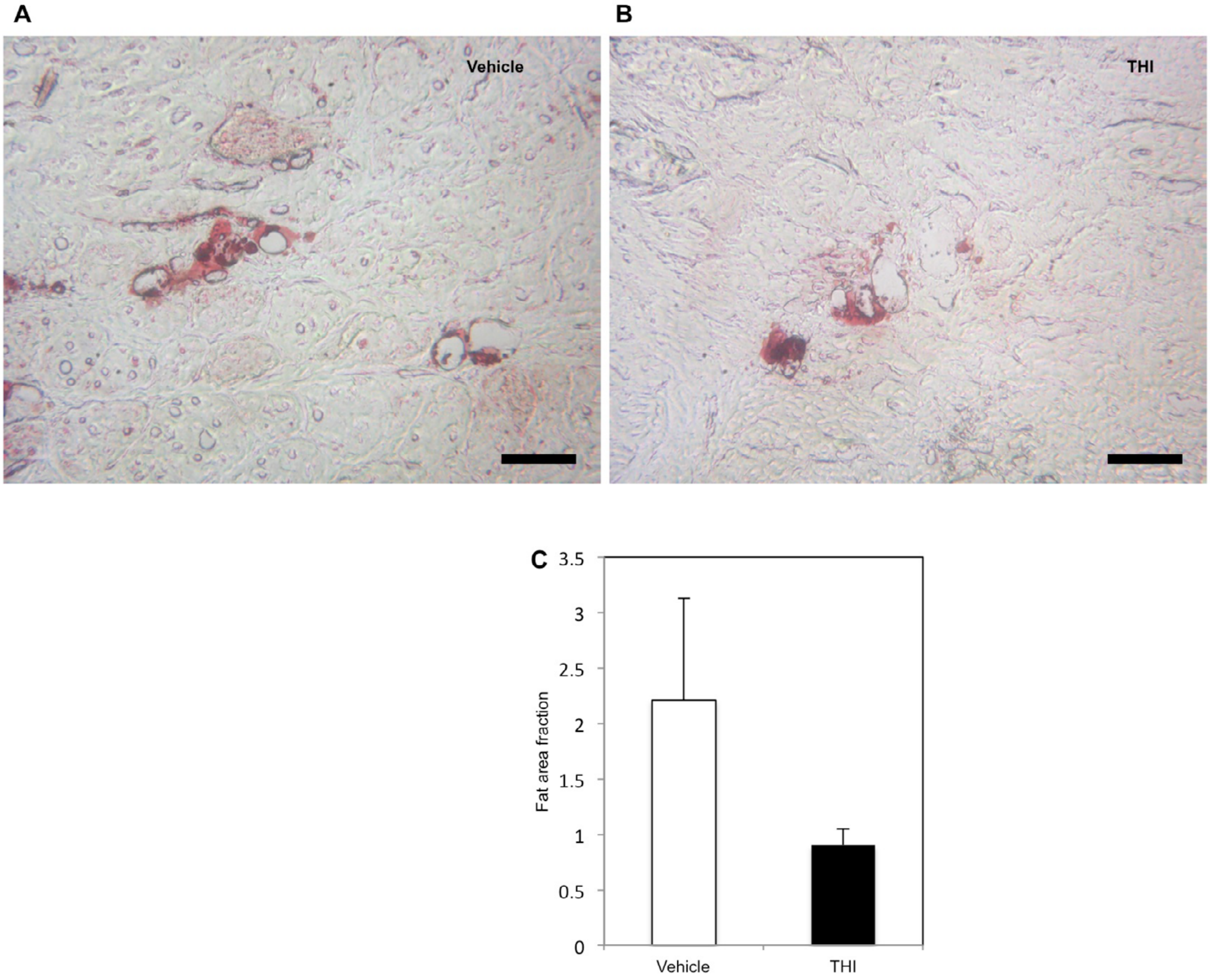


**Fig S5. T cell and macrophage infiltration are not significantly altered in *mdx* diaphragm after 1 month THI treatment**. Diaphragms were immuno-stained with CD45 or CD68 for T cell or macrophage markers, respectively. Cells were quantified by counting CD45 positive clusters in the whole diaphragm muscles or by normalizing CD68 positive cells to the number of muscle cells in diaphragm muscles. No significant differences were observed in CD45 and CD68 positive cells between THI and vehicle treated samples.

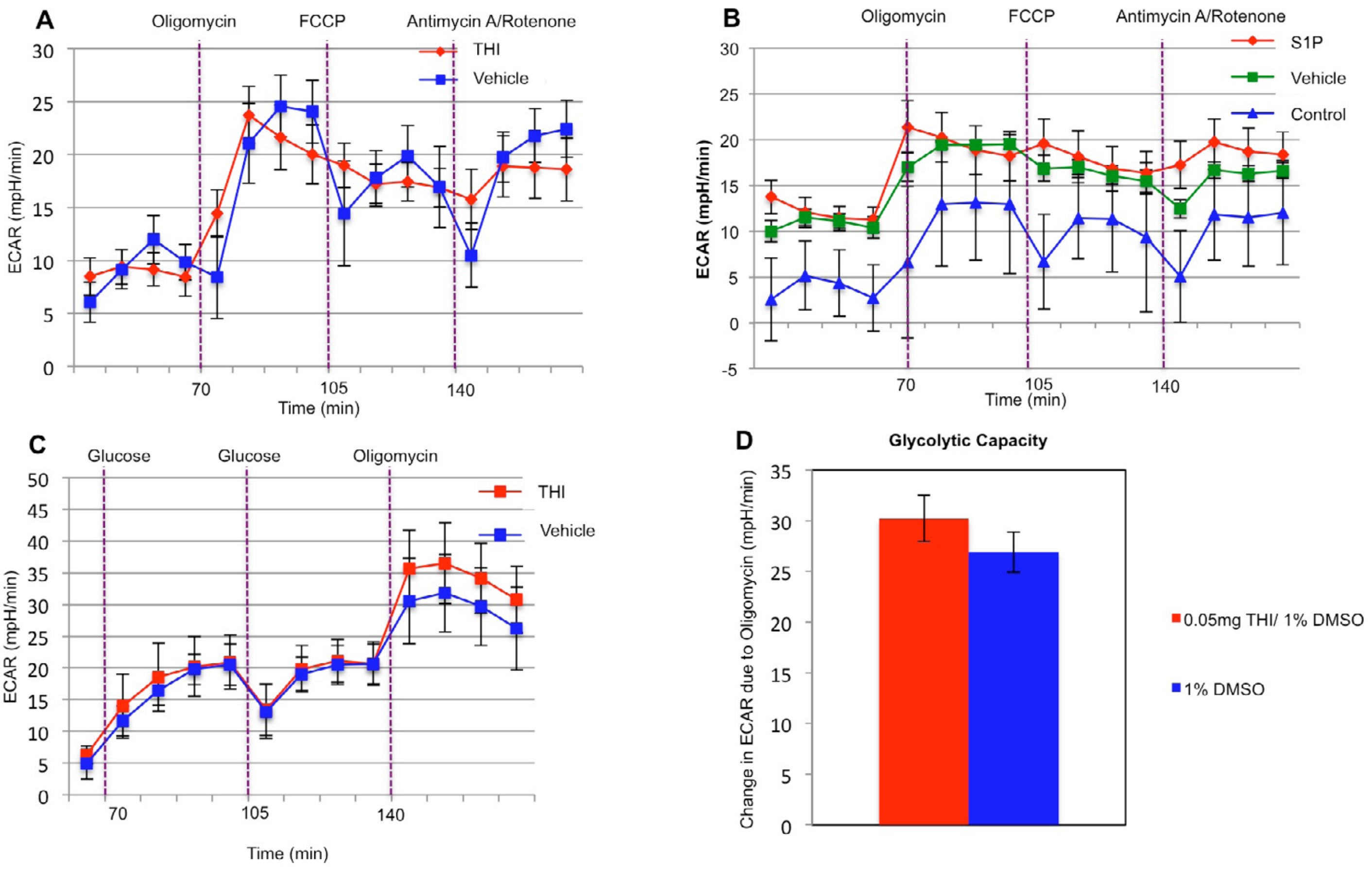


**Fig S6. Extracellular acidification rates in THI or S1P treated, differentiating C2C12 cells do not indicate significant increases in glycolysis.** Seahorse metabolic flux results, normalized to cell number from differentiating C2C12 cells, treated as in Fig. 6. Representative graphs of ECAR changes in response to oligomycine in (A) THI treated, (B) S1P treated, differentiating C2C12. ECAR change graph (C) and quantification (D) of glycolytic capacity test in THI and vehicle treated, differentiating C2C12 (*n*=6 for THI; *n*=6 for vehicle).

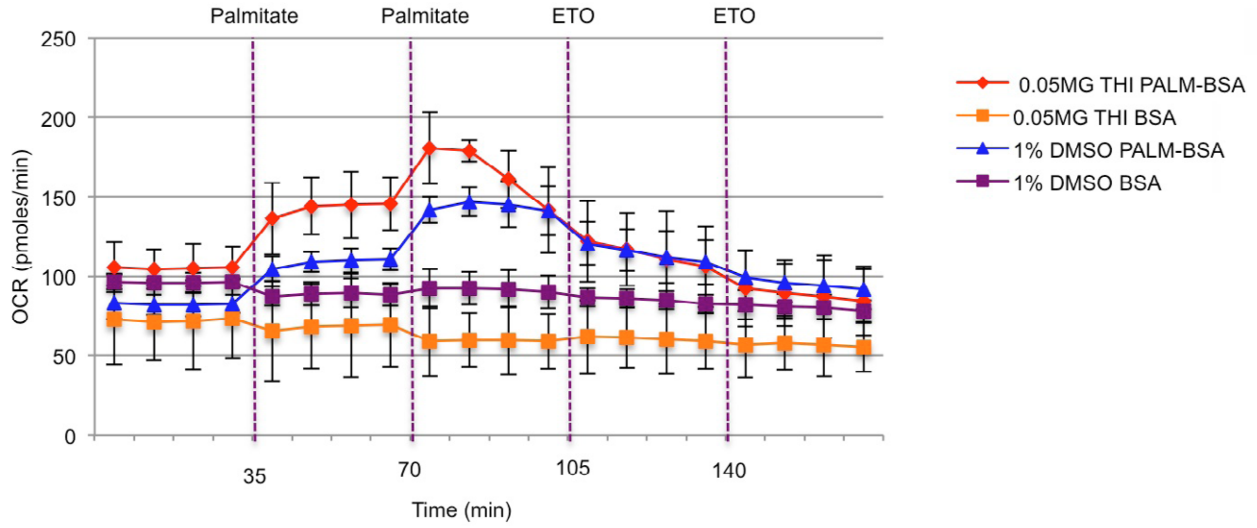


**Fig S7. Representative graph for fatty acid oxidation assay on THI or vehicle treated, differentiating C2C12 cells.**
